# Supplementary material for: Applying High-Value Care Principles in a Pediatric Case: A Workshop for Health Professions Students
Source: MedEdPORTAL. 2020 Nov 17;16:11030. doi: 10.15766/mep_2374-8265.11030 (PMC7678025; doi:10.15766/mep_2374-8265.11030)
Supplement: Supplementary file 1 — Facilitator Guide.docxClinical Vignette.docxPowerPoint Presentation.pptxCost List.xlsxRole-Play Cases.docxPre- and Postsurvey.docx [file mep_2374-8265.11030-s001.zip › A. Facilitator Guide.docx]

**Facilitator Guide- Appendix A**

***Applying high value care principles in a pediatric case: A workshop for medical students***

**Logistics**

Time required: 1.0 hour

**Learners**

The intended audience for this session is medical students, particularly while on pediatric clerkship. A small group setting (9-10 learners per session) is ideal.

**Equipment Needed**:

1. Computer with screen for PowerPoint presentation (suggested)
2. White board or flip chart and colored markers for recording group work (suggested)
3. Timer (recommended but not required)

**Materials**

1. Instructor Guide with timeline and references
2. Lecture slides
3. Clinical case
4. Cost sheet
5. Role-playing cases
6. Pre- and post-surveys, which the facilitator may use to allow students to self-evaluate their own knowledge and the workshop’s effectiveness.

**Workshop Components: Teaching Methods**

1. **Case presentation with small group discussion**

Students are asked to break in to groups of 3 or 4 to discuss the case provided. Each group generates a list of differential diagnoses, initial diagnostic and management plan. Students are not initially told that the topic of the session is high value care. We suggest an alternate title for the presentation, such as Management of the Pediatric Patient, or another option that does not allude to high value care.

1. **Large group debrief**

Each small group provides their top 3 differential diagnoses (written on the white board in a different color per group). After each group has shared their top 3, the facilitator can open the discussion to all groups to add until all possibilities have been shared. Facilitator should not give feedback on accuracy of differential diagnoses at this point. Facilitator then prompts each group to share initial diagnostic plan (ideally in reverse order of differential sharing). Facilitator should ask “what does this help you rule in or rule out?” after each test is requested. For example, if group says Chest X-ray to rule out pneumonia, facilitator will draw arrow to pneumonia on differential. If pneumonia was not on original list of differential diagnoses, facilitator should point this out and ask if pneumonia needs to be added. Facilitator should draw arrow to as many diagnoses on the list that are applicable. Facilitator can also start to introduce some ideas of how tests may not change management. For example, if CBC is requested, facilitator can ask if a normal WBC is possible in bacterial infection or if WBC can be elevated in viral illness. Ask if any additional tests are needed (i.e. blood or urine culture). After diagnostic plan has been discussed, facilitator should ask for management plan. Facilitator should ask for specific modalities as applicable- i.e. if student says oxygen, facilitator should ask how student want oxygen delivered; if student says Albuterol, facilitator should ask in what form. Facilitator can also ask leading questions, such as “do you want to add steroids?” or “does this patient need antibiotics?” The case is intended to be vague enough to allow for some discussion around treatment, but is geared towards being bronchiolitis, so ultimately, very few if any diagnostic measures are indicated, and treatment plan should be geared towards bronchiolitis.

1. **Didactic slide presentation**

When discussing cost of healthcare, can distribute cost sheet as a reference (alternatively, can have each group tally spending for their own diagnostic and management plan)

1. **Practice role playing with debrief**

Learners participate in 1 or 2 role-playing cases. Working in dyads, one is the “parent” and the other is the healthcare professional. The group comes together to debrief as a whole with the facilitator prompting for difficult encounters and suggestions for future encounters. The “parent” role also expresses if they felt their concerns were appropriately addressed. If time permits, learners may switch roles and role-play the 2^nd^ case provided.

1. **Summary and self-evaluation**

Learners have the option to complete a brief survey rating their understanding of high value care and whether the workshop would impact future decision making.

**Description**

This resource is a PowerPoint-based interactive workshop discussing high value care, Choosing Wisely guidelines in Pediatrics, and how to communicate with parents and patients about this topic. The “Choosing Wisely” campaign, initially an initiative of the ABIM Foundation which has been adopted by 50 specialty societies, will be used as the basis of this workshop. The principles of this campaign as well as specific recommendations within Pediatrics will be introduced to learners in order to encourage them to think and talk about medical tests and procedures that may be unnecessary and that in some instances can cause harm.

This workshop introduces the topic of value added care initially by eliciting tests that students want to order naturally and highlighting the concept of whether or not a test changes the management of the patient.

This workshop aims to increase medical students’ confidence in their ability to provide high-value care.

**Educational Objectives**

1. Describe high value cost-conscious care.
2. Analyze cost to the national health system, as well as cost to the patient, when making diagnostic and therapeutic decisions in order to decrease unnecessary healthcare spending.
3. Examine harms and non-monetary costs when making medical decisions.
4. Develop strategies to communicate with patients and families in navigating high-value care discussions.

**Outline for 1-hour workshop**

- - - 1. **Case presentation with small group discussion (10 mins)**
      2. **Large group debrief (10 mins)**
      3. **Powerpoint: Introduction of “Choosing Wisely” Campaign and high value care principles (20 mins)**
      4. **Practice role playing with debrief (10 mins)**
      5. **Summary and self-evaluation (10 mins)**

**Suggested Resources**

1. Free access ABIM “Choosing Wisely” Communication Modules with video example: <http://www.choosingwisely.org/resources/modules/>. Each module has free patient handouts from specialty societies and C*onsumer Reports*.

2. Free access *Consumer Reports* patient handouts: <http://www.consumerreports.org/cro/2012/04/choosing-wisely-how-to-avoid-unnecessary-tests-and-treatments/index.htm>

3. Costs of care: <http://www.costsofcare.org/>

4. High value care pediatric curriculum: Woods S, Avery C, Bartlett K, et al. High value care pediatric curriculum. *MedEdPORTAL*. 2015;11:10146.

https://www.mededportal.org/publication/10146/#322026
