# Supplementary material for: Applying High-Value Care Principles in a Pediatric Case: A Workshop for Health Professions Students
Source: MedEdPORTAL. 2020 Nov 17;16:11030. doi: 10.15766/mep_2374-8265.11030 (PMC7678025; doi:10.15766/mep_2374-8265.11030)
Supplement: Supplementary file 1 — Facilitator Guide.docxClinical Vignette.docxPowerPoint Presentation.pptxCost List.xlsxRole-Play Cases.docxPre- and Postsurvey.docx [file mep_2374-8265.11030-s001.zip › B. Clinical Vignette.docx]

**PATIENT MANAGEMENT CASE- Appendix B**

**Making the diagnosis and treating the patient**

Pediatrics Clerkship

You are working in the Emergency Room and you pick up your next chart. The patient (Jack) is a 14 month old male. He is presenting with his second episode of difficulty breathing.

He presents to the ED with 4 days of cough and congestion. Yesterday he felt warm and he started having difficulty breathing. He hasn’t been eating well over the past several days and vomited twice after coughing. He has had no diarrhea and his last bowel movement was 2 days ago. He has no rash. He hasn’t been as playful as usual but he will interact with his brother. His older brother has had a cough as well, but not as bad as Jack’s. No one else has been sick at home. Mom is really concerned about his cough and wants to know what you can give for it. She also read on Wikipedia that this is the exact same presentation as a child who died of MRSA pneumonia. That really concerns her too.

PMH: mild eczema, immunizations up to date except for Flu

On examination, Jack is awake but looks uncomfortable. His temperature is 99.3°F. He is breathing at 65 breaths per minute and his oxygen saturation is 88% on room air. He has copious nasal secretions and his nostrils are flaring but there is no stridor. He has subcostal, intercostal and supraclavicular retractions and diffuse coarse crackles bilaterally with no wheezing. His heart exam is normal. His abdomen is a little distended but there are no masses. His feet are warm and his cap refill is less than 2 seconds.

Mom wants to know what Jack’s diagnosis is and what you are going to do about it.

Your mission, should you choose to accept it, is to decide what diagnostic measures you would like and what treatment modalities you would like.
