# Supplementary material for: Applying High-Value Care Principles in a Pediatric Case: A Workshop for Health Professions Students
Source: MedEdPORTAL. 2020 Nov 17;16:11030. doi: 10.15766/mep_2374-8265.11030 (PMC7678025; doi:10.15766/mep_2374-8265.11030)
Supplement: Supplementary file 1 — Facilitator Guide.docxClinical Vignette.docxPowerPoint Presentation.pptxCost List.xlsxRole-Play Cases.docxPre- and Postsurvey.docx [file mep_2374-8265.11030-s001.zip › C. PowerPoint Presentation.pptx]

## Slide 1
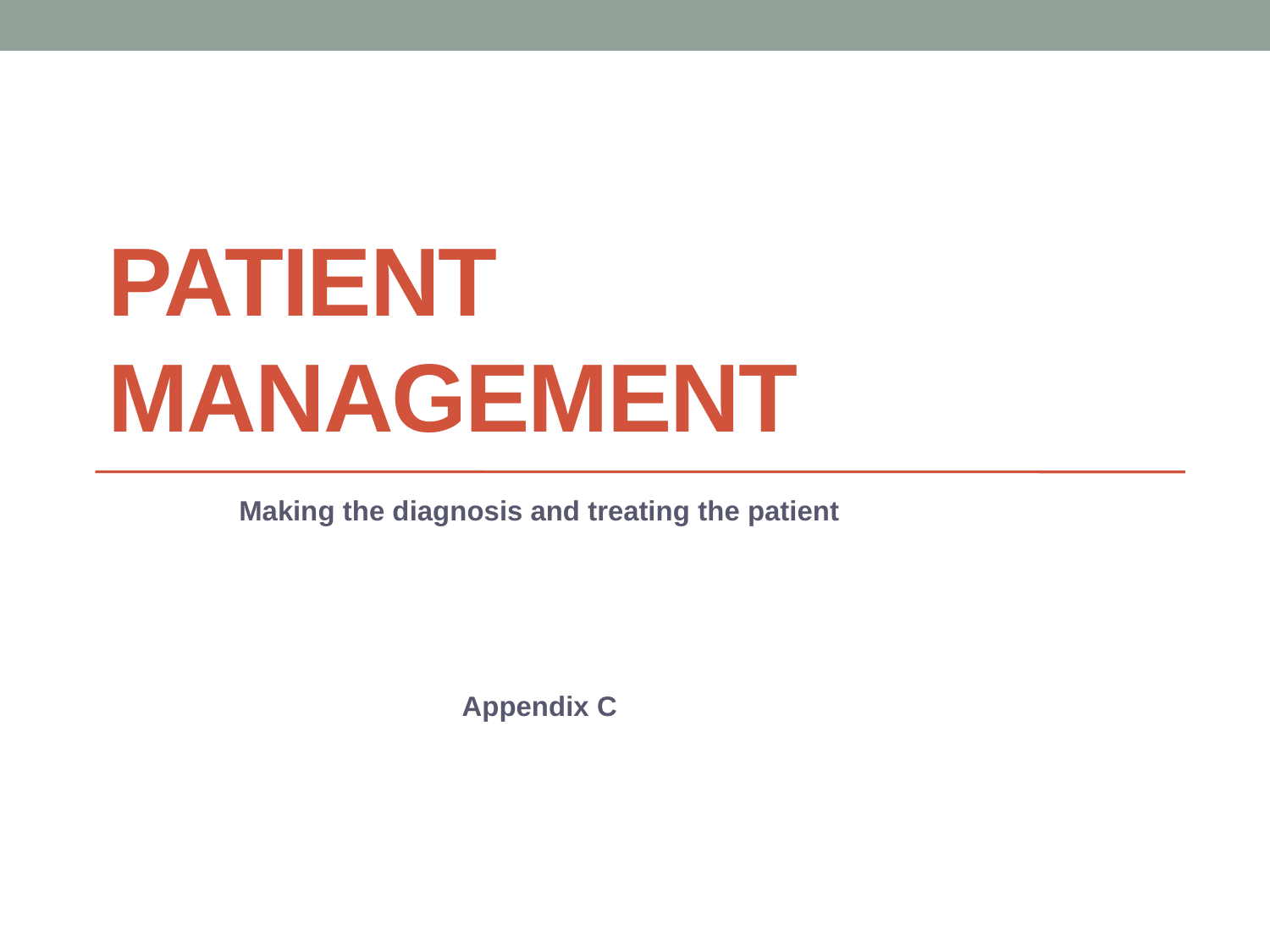

# Patient Management
Making the diagnosis and treating the patient
Appendix C

## Slide 2
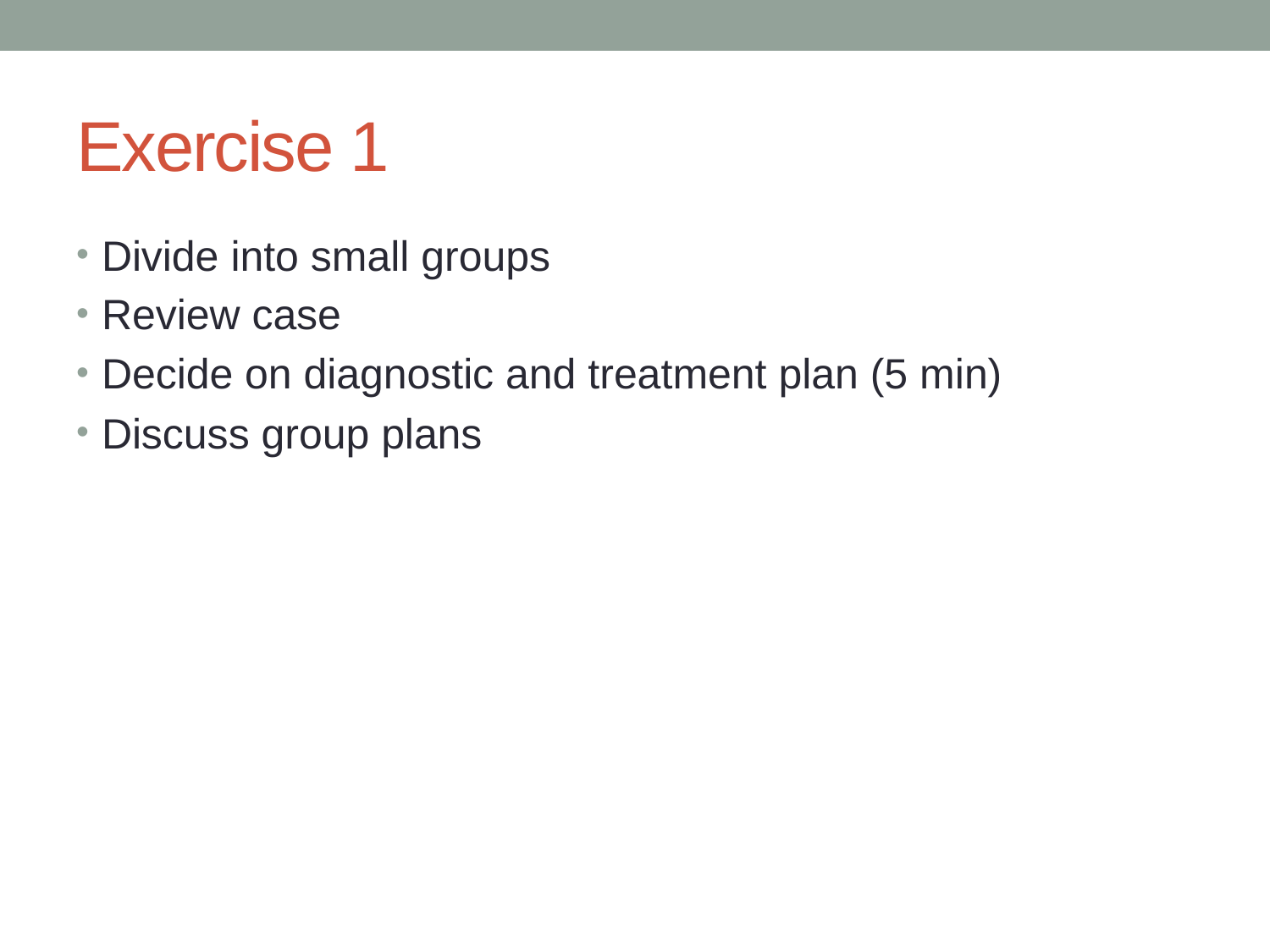

# Exercise 1
Divide into small groups
Review case
Decide on diagnostic and treatment plan (5 min)
Discuss group plans

## Slide 3
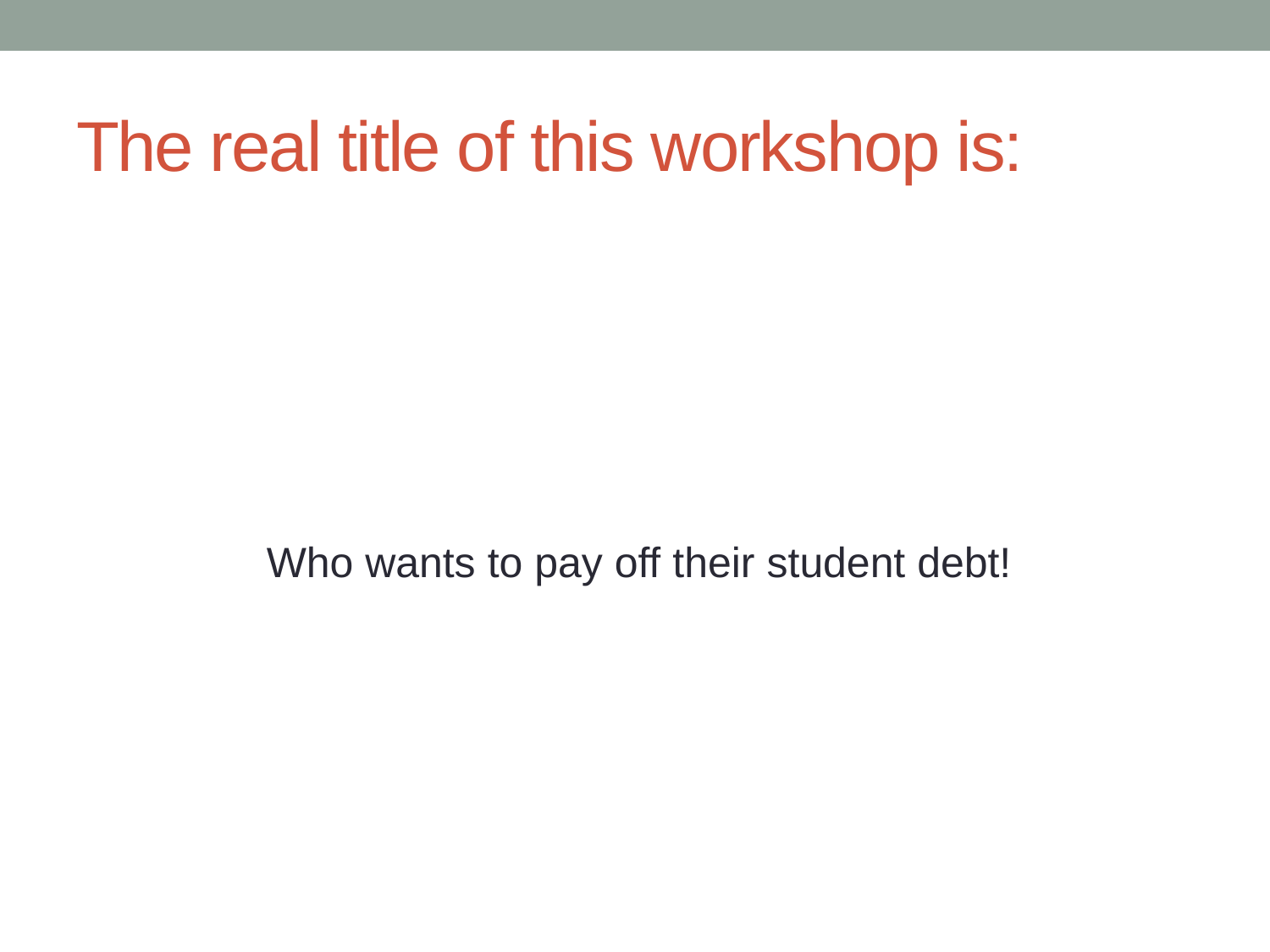

# The real title of this workshop is:
Who wants to pay off their student debt!

## Slide 4
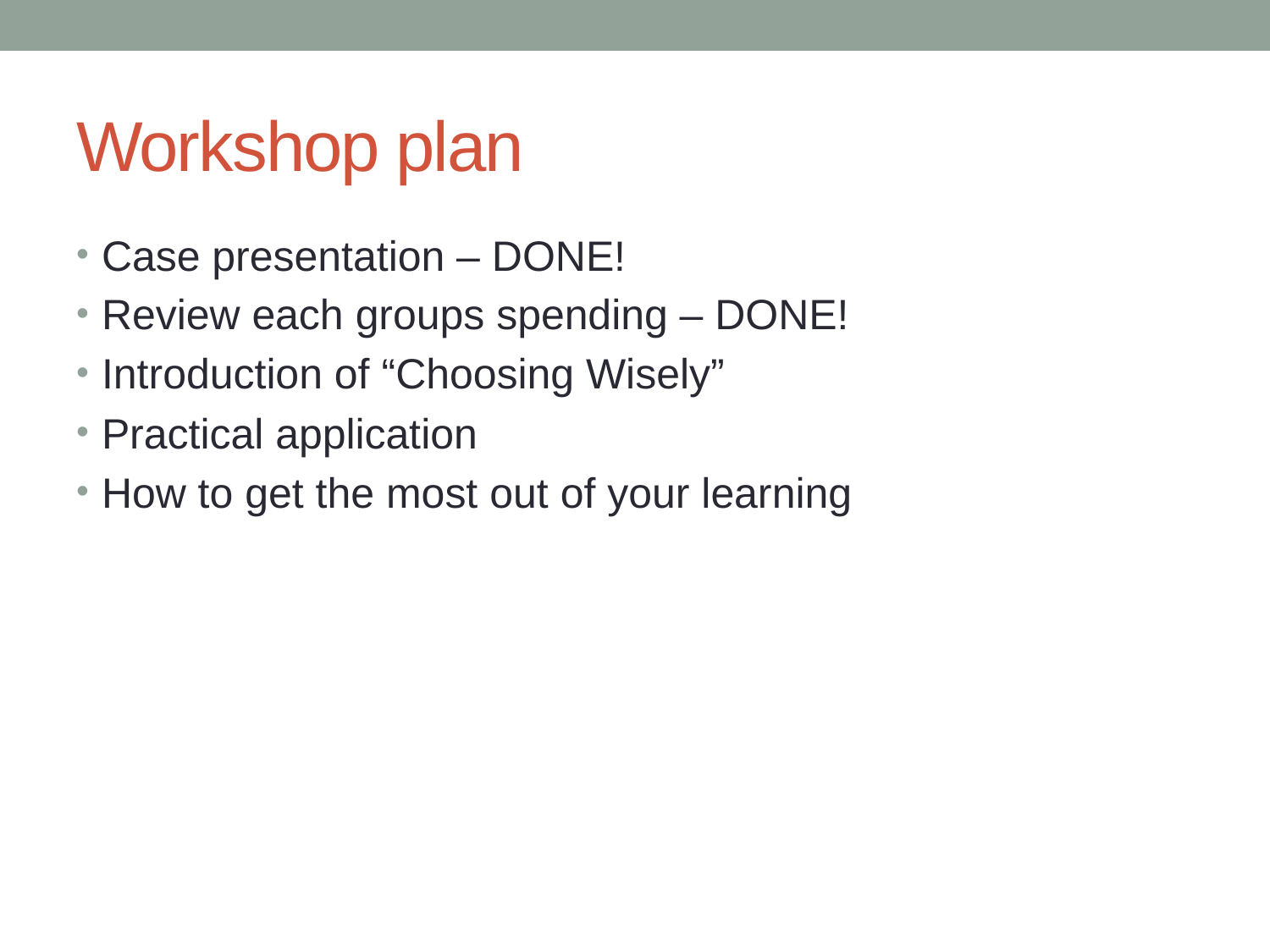

# Workshop plan
Case presentation – DONE!
Review each groups spending – DONE!
Introduction of “Choosing Wisely”
Practical application
How to get the most out of your learning

## Slide 5
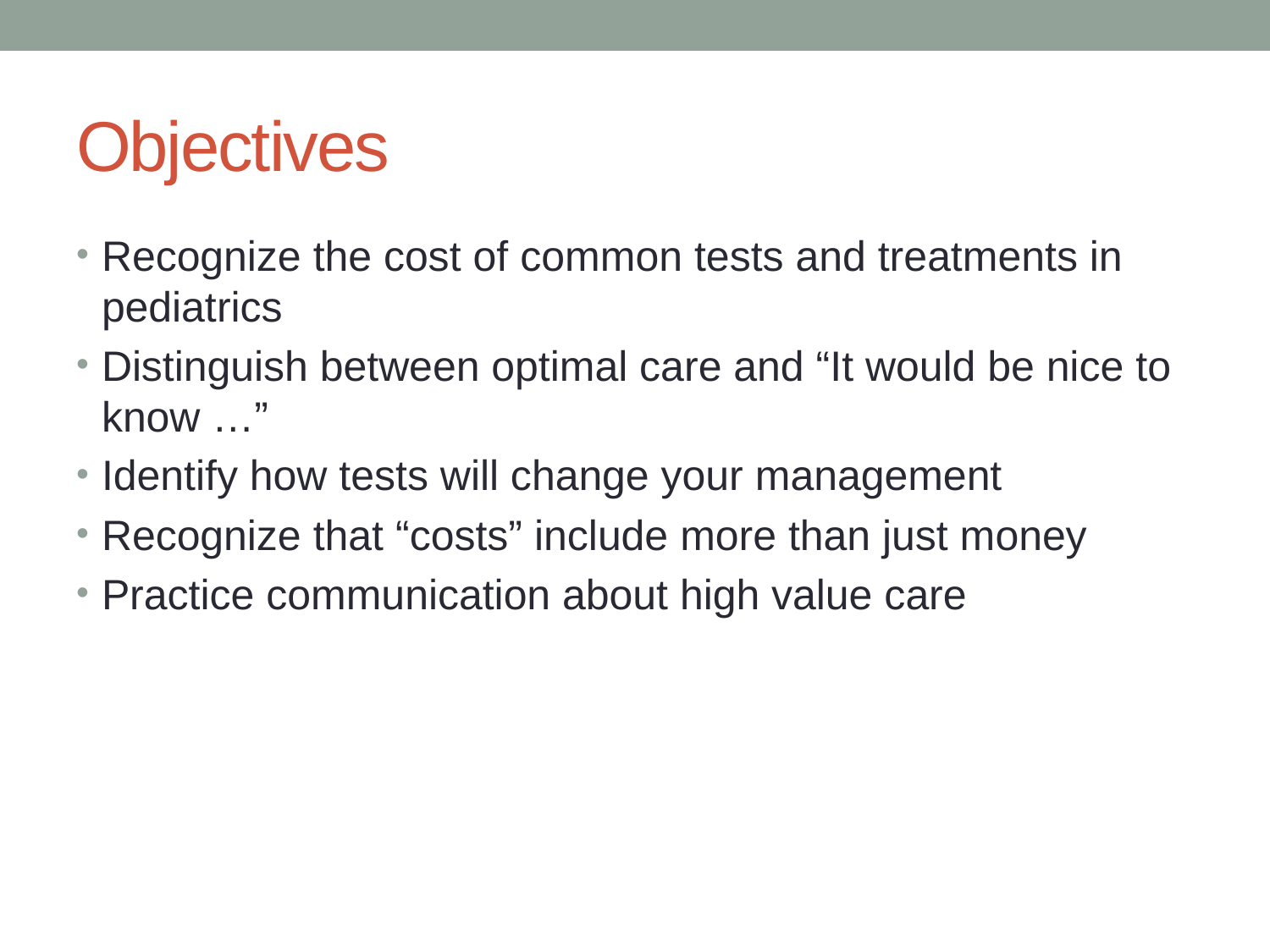

# Objectives
Recognize the cost of common tests and treatments in pediatrics
Distinguish between optimal care and “It would be nice to know …”
Identify how tests will change your management
Recognize that “costs” include more than just money
Practice communication about high value care

## Slide 6
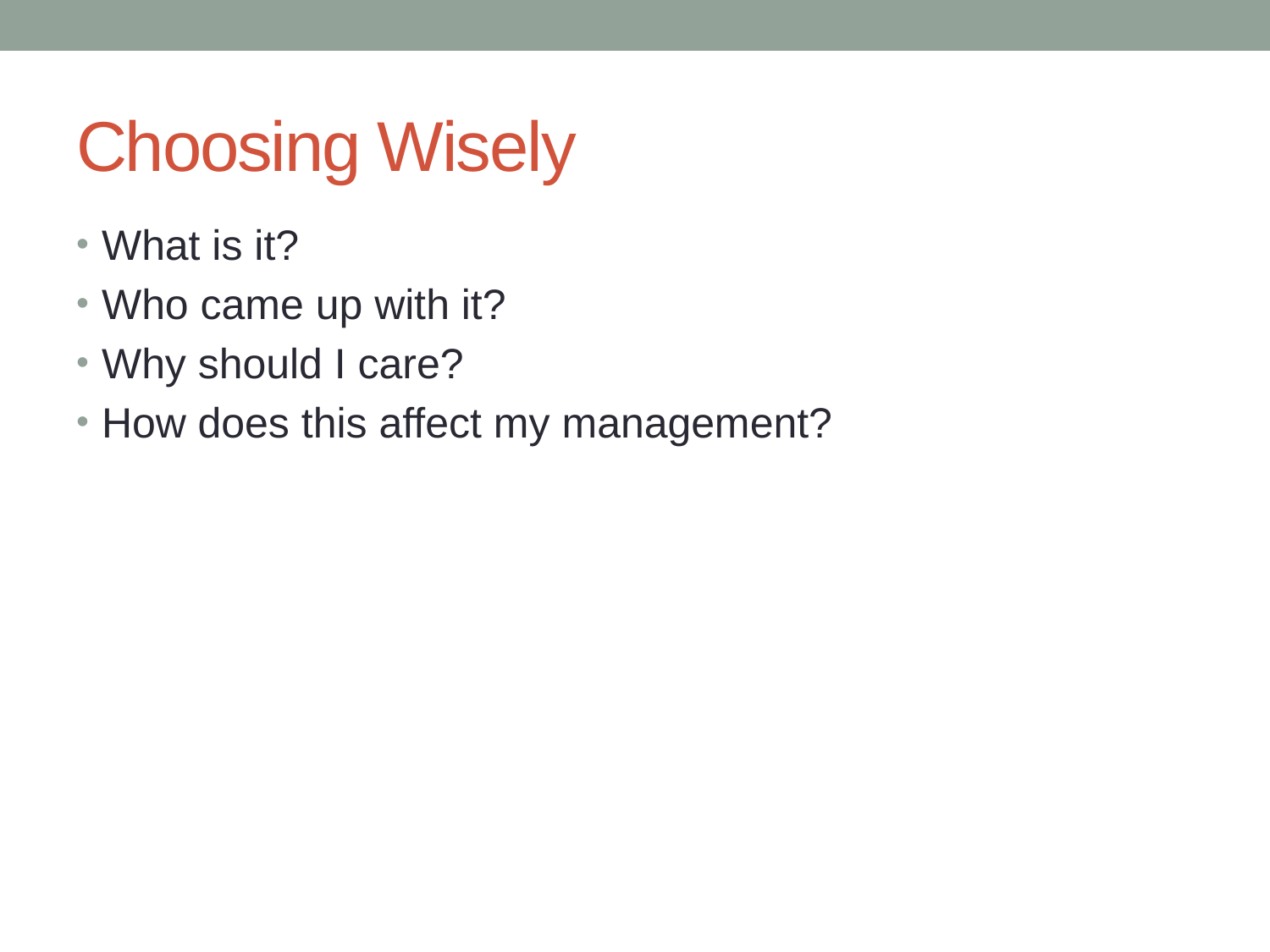

# Choosing Wisely
What is it?
Who came up with it?
Why should I care?
How does this affect my management?

## Slide 7
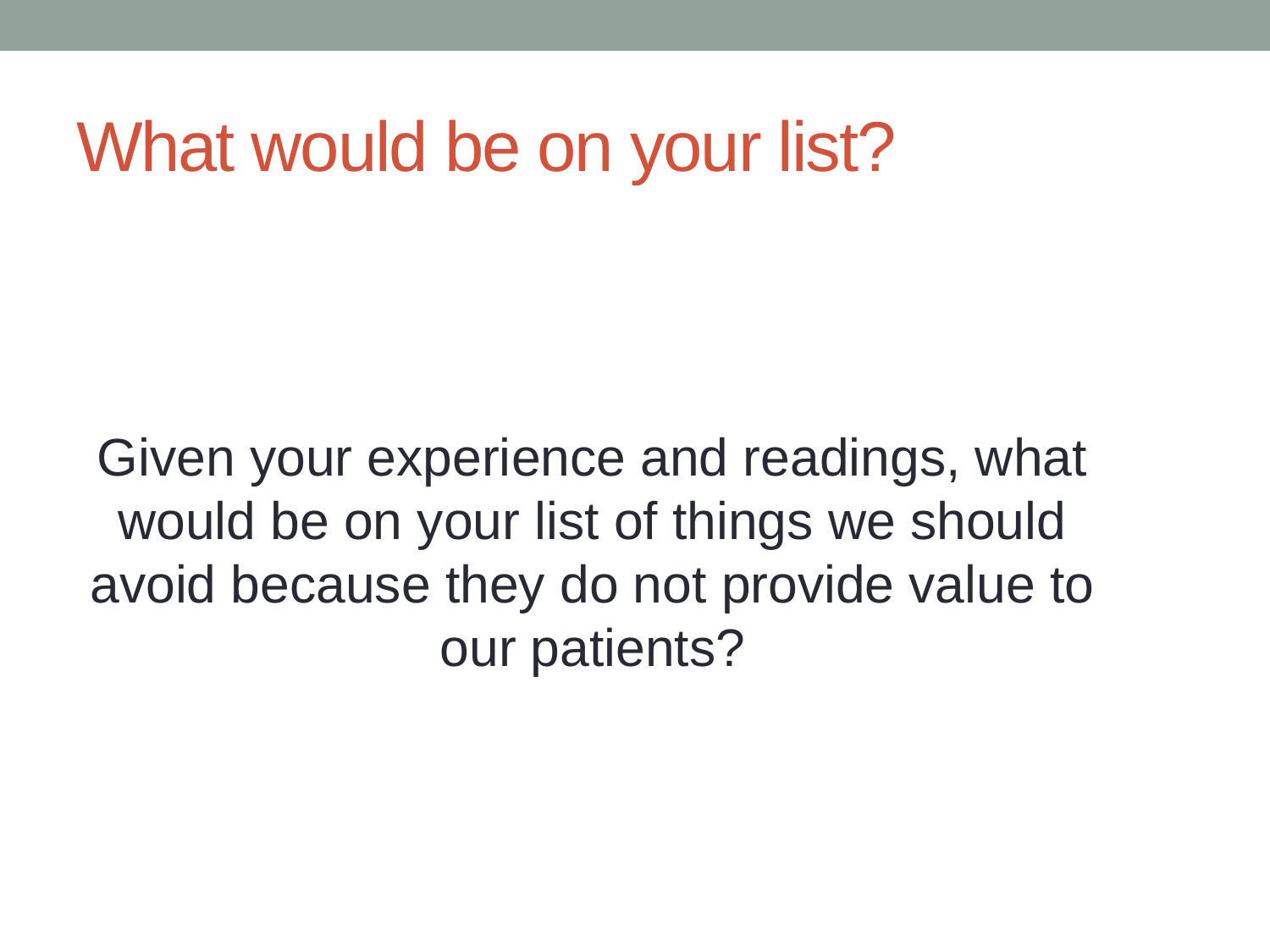

# What would be on your list?
Given your experience and readings, what would be on your list of things we should avoid because they do not provide value to our patients?

## Slide 8
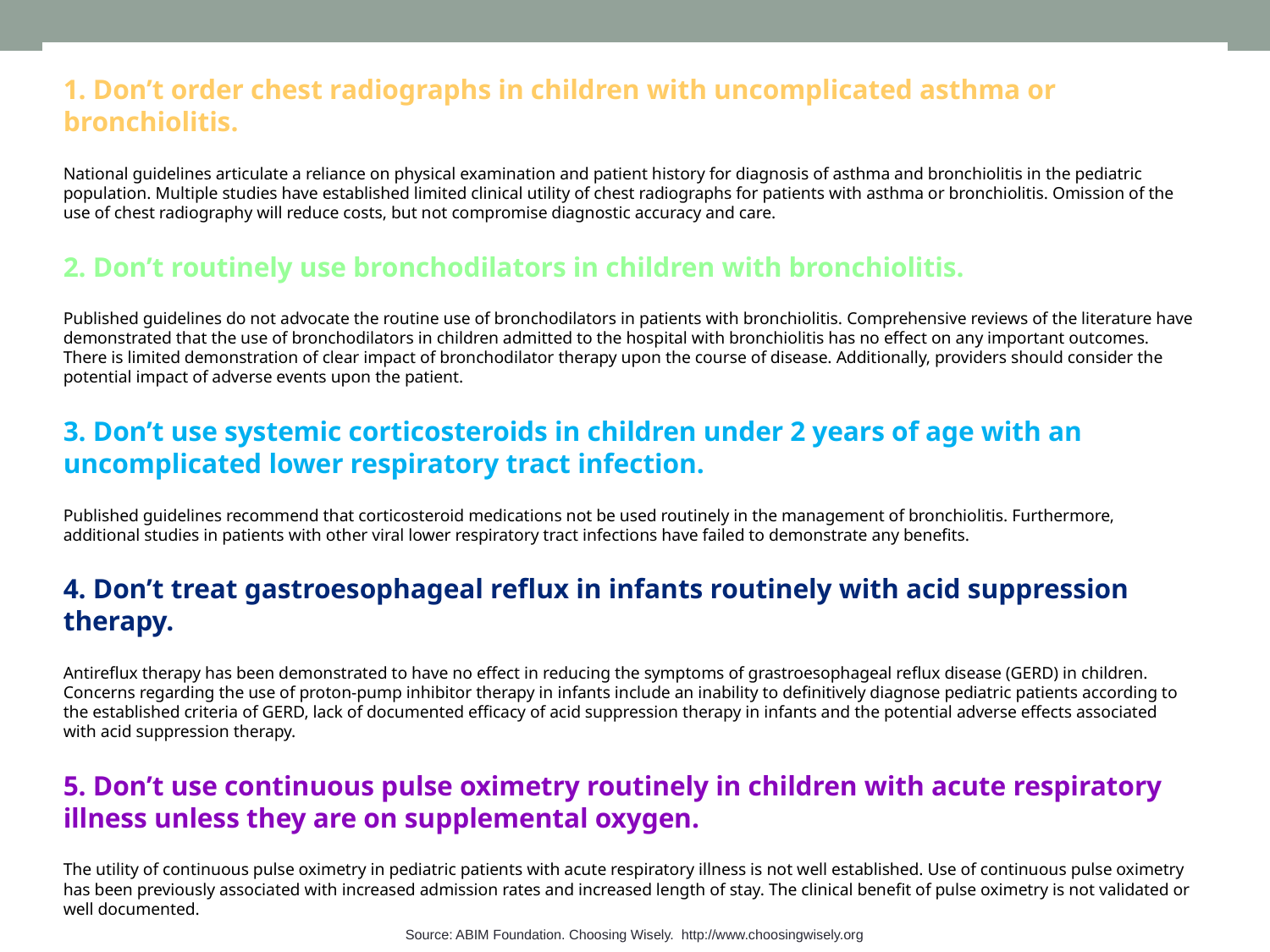

1. Don’t order chest radiographs in children with uncomplicated asthma or bronchiolitis.
National guidelines articulate a reliance on physical examination and patient history for diagnosis of asthma and bronchiolitis in the pediatric population. Multiple studies have established limited clinical utility of chest radiographs for patients with asthma or bronchiolitis. Omission of the use of chest radiography will reduce costs, but not compromise diagnostic accuracy and care.
2. Don’t routinely use bronchodilators in children with bronchiolitis.
Published guidelines do not advocate the routine use of bronchodilators in patients with bronchiolitis. Comprehensive reviews of the literature have demonstrated that the use of bronchodilators in children admitted to the hospital with bronchiolitis has no effect on any important outcomes. There is limited demonstration of clear impact of bronchodilator therapy upon the course of disease. Additionally, providers should consider the potential impact of adverse events upon the patient.
3. Don’t use systemic corticosteroids in children under 2 years of age with an uncomplicated lower respiratory tract infection.
Published guidelines recommend that corticosteroid medications not be used routinely in the management of bronchiolitis. Furthermore, additional studies in patients with other viral lower respiratory tract infections have failed to demonstrate any benefits.
4. Don’t treat gastroesophageal reflux in infants routinely with acid suppression therapy.
Antireflux therapy has been demonstrated to have no effect in reducing the symptoms of grastroesophageal reflux disease (GERD) in children. Concerns regarding the use of proton-pump inhibitor therapy in infants include an inability to definitively diagnose pediatric patients according to the established criteria of GERD, lack of documented efficacy of acid suppression therapy in infants and the potential adverse effects associated with acid suppression therapy.
5. Don’t use continuous pulse oximetry routinely in children with acute respiratory illness unless they are on supplemental oxygen.
The utility of continuous pulse oximetry in pediatric patients with acute respiratory illness is not well established. Use of continuous pulse oximetry has been previously associated with increased admission rates and increased length of stay. The clinical benefit of pulse oximetry is not validated or well documented.
Source: ABIM Foundation. Choosing Wisely. http://www.choosingwisely.org

## Slide 9
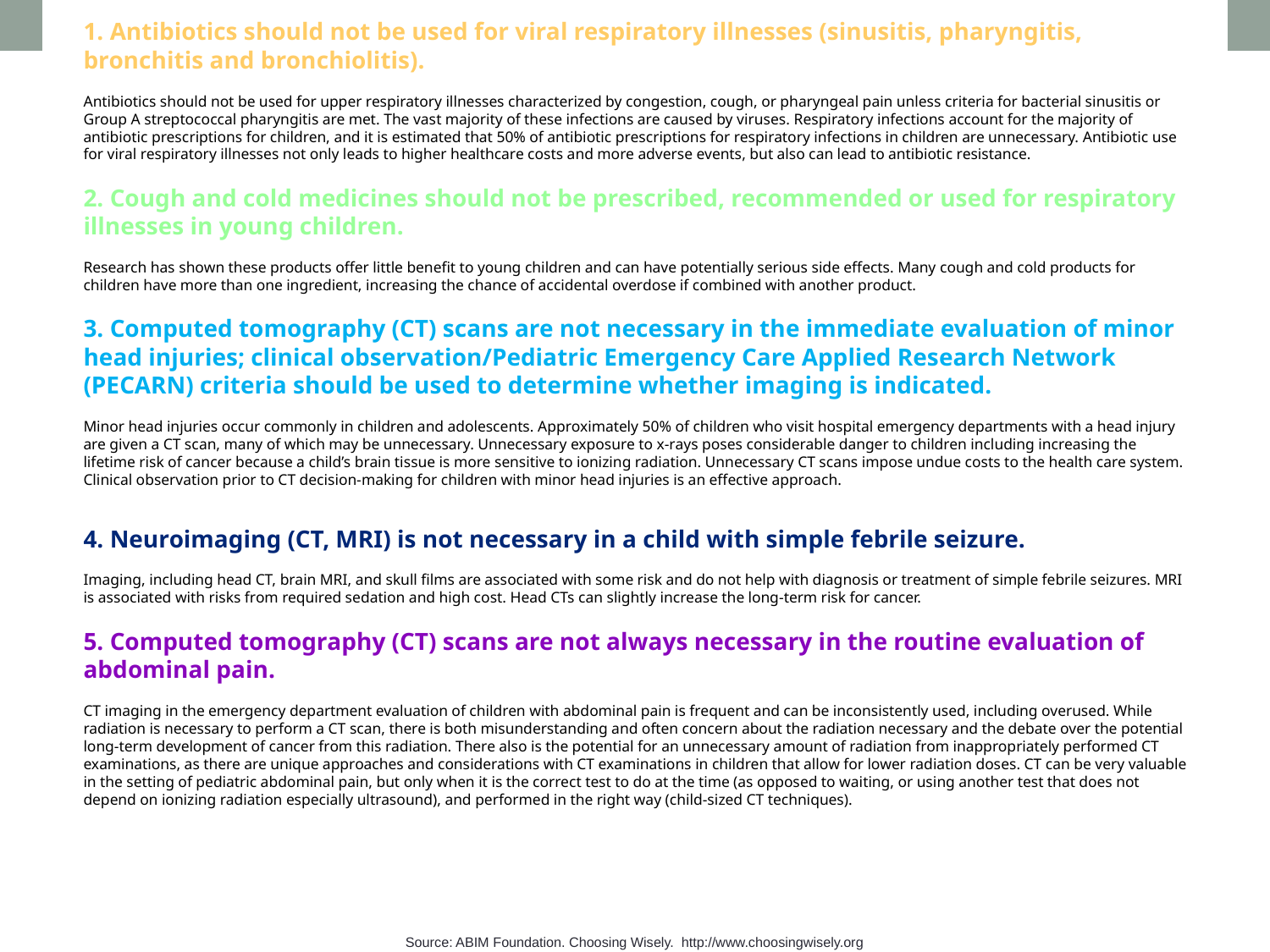

1. Antibiotics should not be used for viral respiratory illnesses (sinusitis, pharyngitis, bronchitis and bronchiolitis).
Antibiotics should not be used for upper respiratory illnesses characterized by congestion, cough, or pharyngeal pain unless criteria for bacterial sinusitis or Group A streptococcal pharyngitis are met. The vast majority of these infections are caused by viruses. Respiratory infections account for the majority of antibiotic prescriptions for children, and it is estimated that 50% of antibiotic prescriptions for respiratory infections in children are unnecessary. Antibiotic use for viral respiratory illnesses not only leads to higher healthcare costs and more adverse events, but also can lead to antibiotic resistance.
2. Cough and cold medicines should not be prescribed, recommended or used for respiratory illnesses in young children.
Research has shown these products offer little benefit to young children and can have potentially serious side effects. Many cough and cold products for children have more than one ingredient, increasing the chance of accidental overdose if combined with another product.
3. Computed tomography (CT) scans are not necessary in the immediate evaluation of minor head injuries; clinical observation/Pediatric Emergency Care Applied Research Network (PECARN) criteria should be used to determine whether imaging is indicated.
Minor head injuries occur commonly in children and adolescents. Approximately 50% of children who visit hospital emergency departments with a head injury are given a CT scan, many of which may be unnecessary. Unnecessary exposure to x-rays poses considerable danger to children including increasing the lifetime risk of cancer because a child’s brain tissue is more sensitive to ionizing radiation. Unnecessary CT scans impose undue costs to the health care system. Clinical observation prior to CT decision-making for children with minor head injuries is an effective approach.
4. Neuroimaging (CT, MRI) is not necessary in a child with simple febrile seizure.
Imaging, including head CT, brain MRI, and skull films are associated with some risk and do not help with diagnosis or treatment of simple febrile seizures. MRI is associated with risks from required sedation and high cost. Head CTs can slightly increase the long-term risk for cancer.
5. Computed tomography (CT) scans are not always necessary in the routine evaluation of abdominal pain.
CT imaging in the emergency department evaluation of children with abdominal pain is frequent and can be inconsistently used, including overused. While radiation is necessary to perform a CT scan, there is both misunderstanding and often concern about the radiation necessary and the debate over the potential long-term development of cancer from this radiation. There also is the potential for an unnecessary amount of radiation from inappropriately performed CT examinations, as there are unique approaches and considerations with CT examinations in children that allow for lower radiation doses. CT can be very valuable in the setting of pediatric abdominal pain, but only when it is the correct test to do at the time (as opposed to waiting, or using another test that does not depend on ionizing radiation especially ultrasound), and performed in the right way (child-sized CT techniques).
Source: ABIM Foundation. Choosing Wisely. http://www.choosingwisely.org

## Slide 10
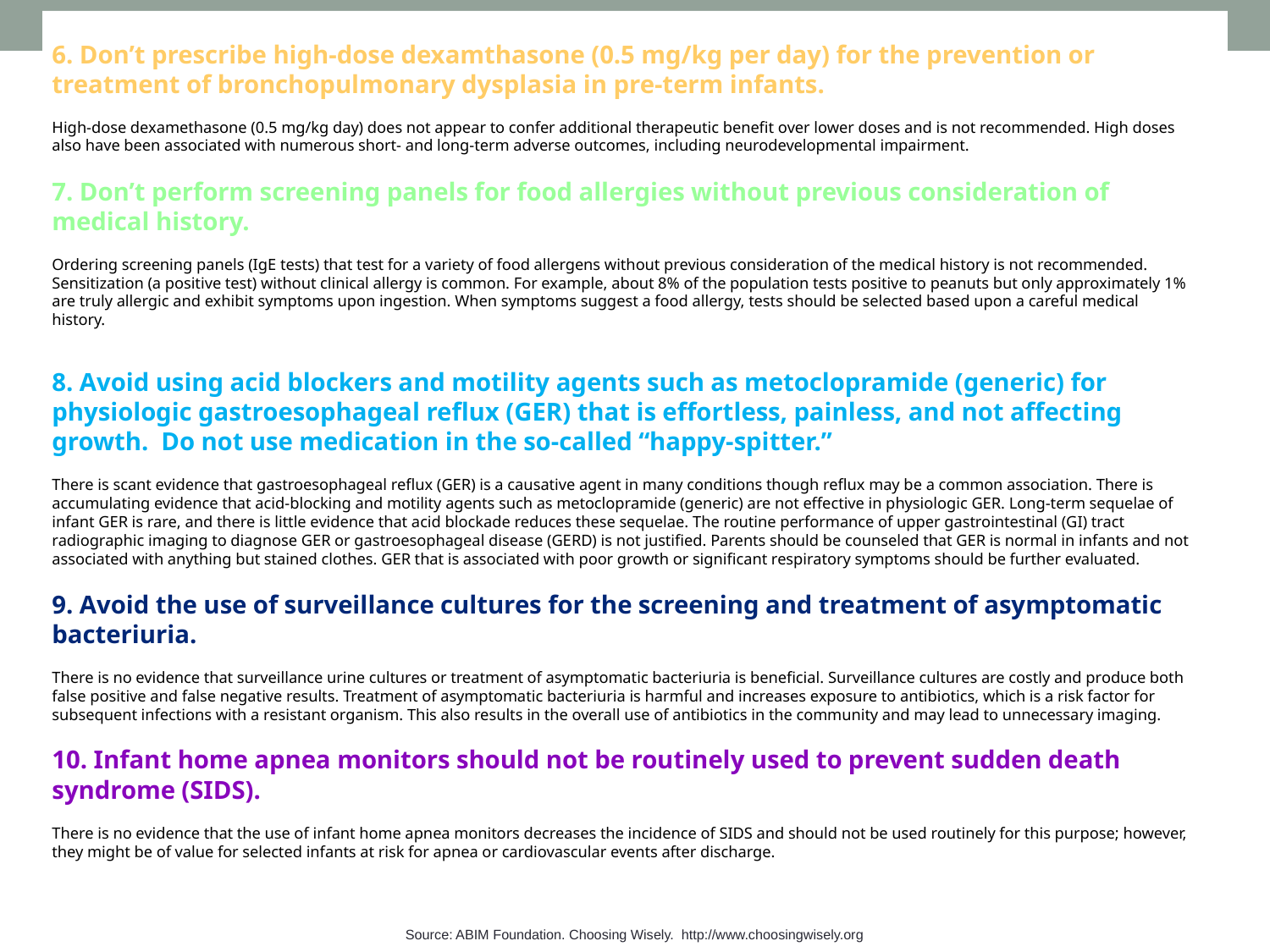

6. Don’t prescribe high-dose dexamthasone (0.5 mg/kg per day) for the prevention or treatment of bronchopulmonary dysplasia in pre-term infants.
High-dose dexamethasone (0.5 mg/kg day) does not appear to confer additional therapeutic benefit over lower doses and is not recommended. High doses also have been associated with numerous short- and long-term adverse outcomes, including neurodevelopmental impairment.
7. Don’t perform screening panels for food allergies without previous consideration of medical history.
Ordering screening panels (IgE tests) that test for a variety of food allergens without previous consideration of the medical history is not recommended. Sensitization (a positive test) without clinical allergy is common. For example, about 8% of the population tests positive to peanuts but only approximately 1% are truly allergic and exhibit symptoms upon ingestion. When symptoms suggest a food allergy, tests should be selected based upon a careful medical history.
8. Avoid using acid blockers and motility agents such as metoclopramide (generic) for physiologic gastroesophageal reflux (GER) that is effortless, painless, and not affecting growth. Do not use medication in the so-called “happy-spitter.”
There is scant evidence that gastroesophageal reflux (GER) is a causative agent in many conditions though reflux may be a common association. There is accumulating evidence that acid-blocking and motility agents such as metoclopramide (generic) are not effective in physiologic GER. Long-term sequelae of infant GER is rare, and there is little evidence that acid blockade reduces these sequelae. The routine performance of upper gastrointestinal (GI) tract radiographic imaging to diagnose GER or gastroesophageal disease (GERD) is not justified. Parents should be counseled that GER is normal in infants and not associated with anything but stained clothes. GER that is associated with poor growth or significant respiratory symptoms should be further evaluated.
9. Avoid the use of surveillance cultures for the screening and treatment of asymptomatic bacteriuria.
There is no evidence that surveillance urine cultures or treatment of asymptomatic bacteriuria is beneficial. Surveillance cultures are costly and produce both false positive and false negative results. Treatment of asymptomatic bacteriuria is harmful and increases exposure to antibiotics, which is a risk factor for subsequent infections with a resistant organism. This also results in the overall use of antibiotics in the community and may lead to unnecessary imaging.
10. Infant home apnea monitors should not be routinely used to prevent sudden death syndrome (SIDS).
There is no evidence that the use of infant home apnea monitors decreases the incidence of SIDS and should not be used routinely for this purpose; however, they might be of value for selected infants at risk for apnea or cardiovascular events after discharge..
Source: ABIM Foundation. Choosing Wisely. http://www.choosingwisely.org

## Slide 11
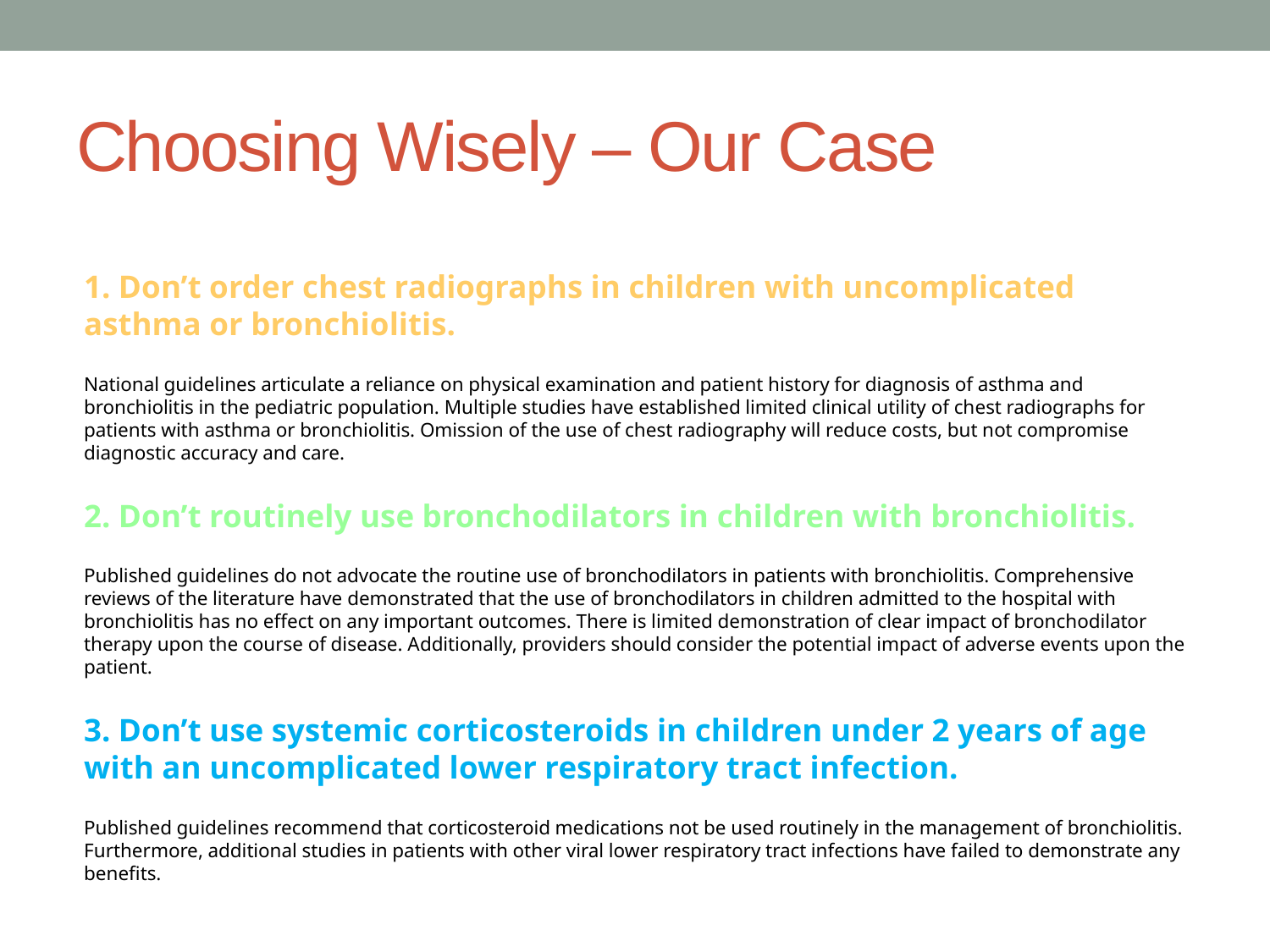

# Choosing Wisely – Our Case
1. Don’t order chest radiographs in children with uncomplicated asthma or bronchiolitis.
National guidelines articulate a reliance on physical examination and patient history for diagnosis of asthma and bronchiolitis in the pediatric population. Multiple studies have established limited clinical utility of chest radiographs for patients with asthma or bronchiolitis. Omission of the use of chest radiography will reduce costs, but not compromise diagnostic accuracy and care.
2. Don’t routinely use bronchodilators in children with bronchiolitis.
Published guidelines do not advocate the routine use of bronchodilators in patients with bronchiolitis. Comprehensive reviews of the literature have demonstrated that the use of bronchodilators in children admitted to the hospital with bronchiolitis has no effect on any important outcomes. There is limited demonstration of clear impact of bronchodilator therapy upon the course of disease. Additionally, providers should consider the potential impact of adverse events upon the patient.
3. Don’t use systemic corticosteroids in children under 2 years of age with an uncomplicated lower respiratory tract infection.
Published guidelines recommend that corticosteroid medications not be used routinely in the management of bronchiolitis. Furthermore, additional studies in patients with other viral lower respiratory tract infections have failed to demonstrate any benefits.

## Slide 12
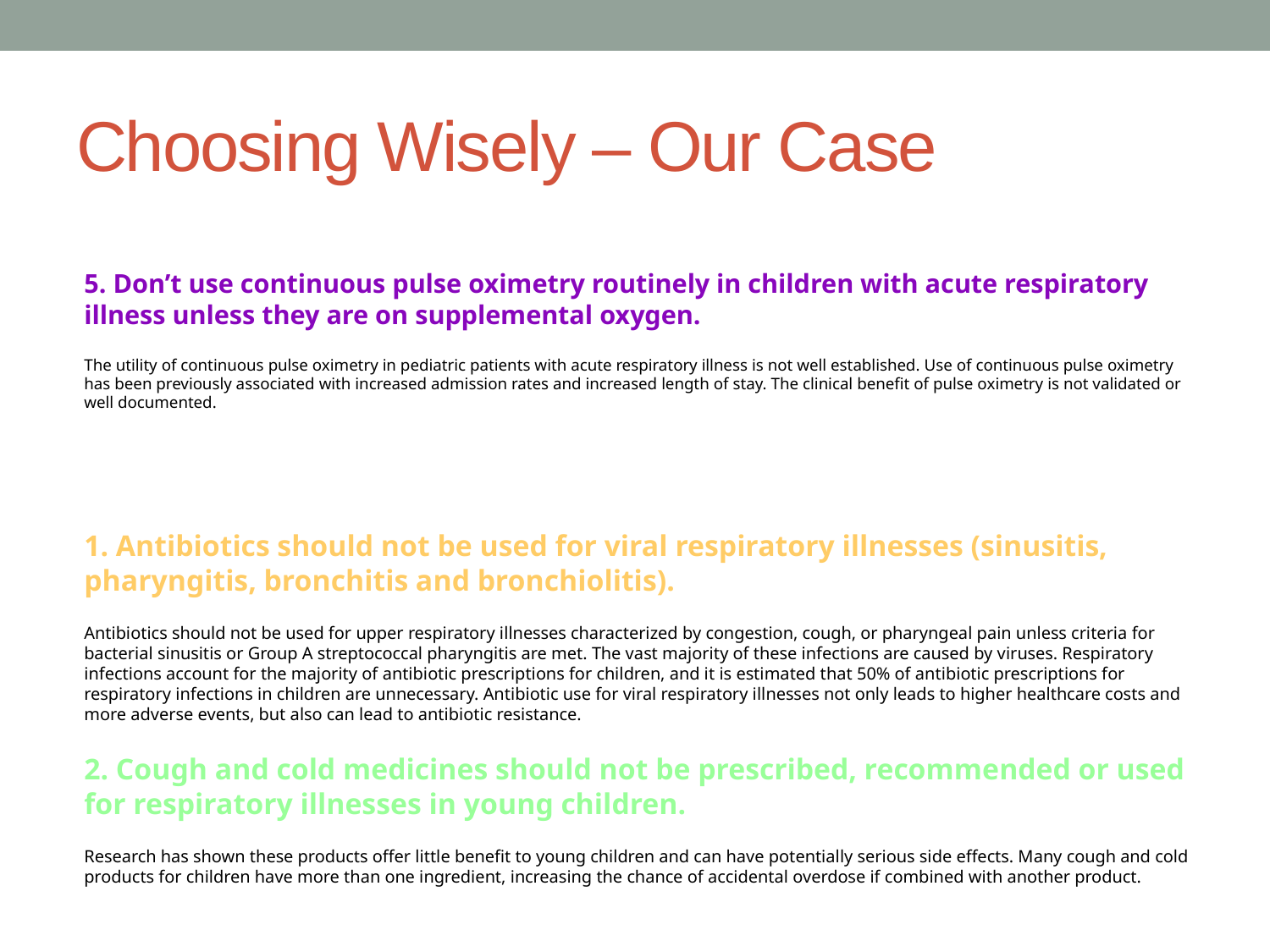

# Choosing Wisely – Our Case
5. Don’t use continuous pulse oximetry routinely in children with acute respiratory illness unless they are on supplemental oxygen.
The utility of continuous pulse oximetry in pediatric patients with acute respiratory illness is not well established. Use of continuous pulse oximetry has been previously associated with increased admission rates and increased length of stay. The clinical benefit of pulse oximetry is not validated or well documented.
1. Antibiotics should not be used for viral respiratory illnesses (sinusitis, pharyngitis, bronchitis and bronchiolitis).
Antibiotics should not be used for upper respiratory illnesses characterized by congestion, cough, or pharyngeal pain unless criteria for bacterial sinusitis or Group A streptococcal pharyngitis are met. The vast majority of these infections are caused by viruses. Respiratory infections account for the majority of antibiotic prescriptions for children, and it is estimated that 50% of antibiotic prescriptions for respiratory infections in children are unnecessary. Antibiotic use for viral respiratory illnesses not only leads to higher healthcare costs and more adverse events, but also can lead to antibiotic resistance.
2. Cough and cold medicines should not be prescribed, recommended or used for respiratory illnesses in young children.
Research has shown these products offer little benefit to young children and can have potentially serious side effects. Many cough and cold products for children have more than one ingredient, increasing the chance of accidental overdose if combined with another product.

## Slide 13
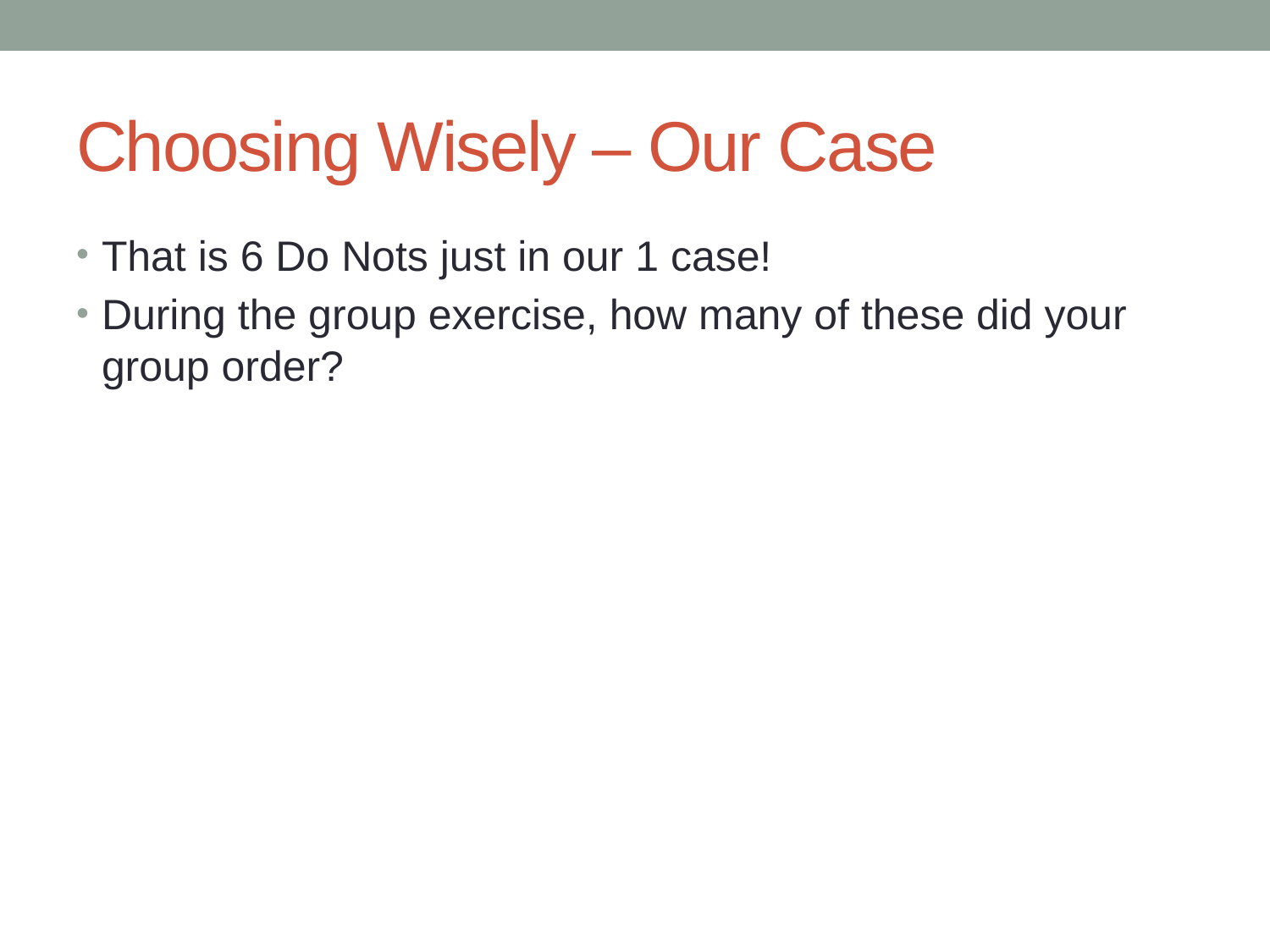

# Choosing Wisely – Our Case
That is 6 Do Nots just in our 1 case!
During the group exercise, how many of these did your group order?

## Slide 14
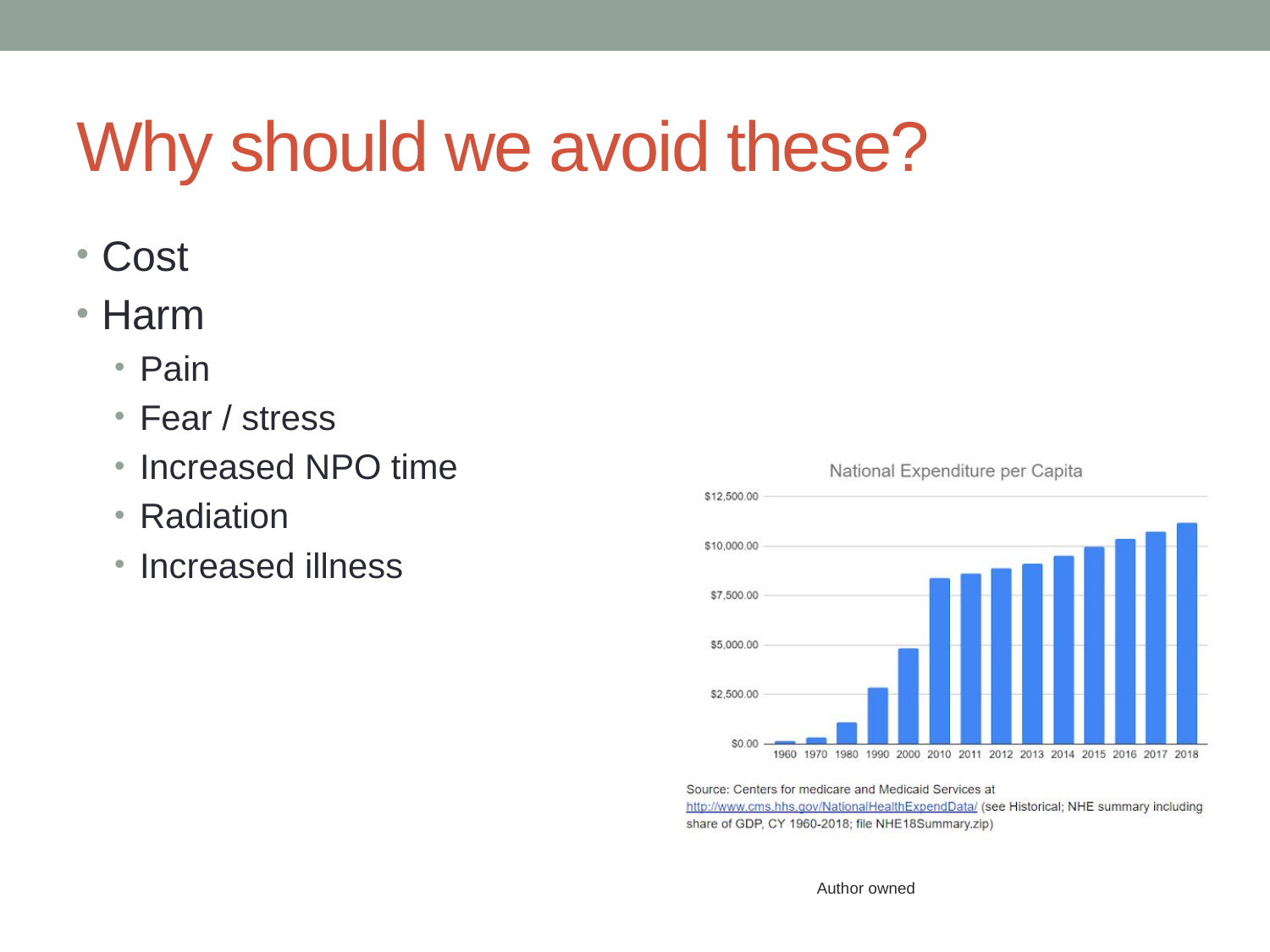

# Why should we avoid these?
Cost
Harm
Pain
Fear / stress
Increased NPO time
Radiation
Increased illness
Author owned

## Slide 15
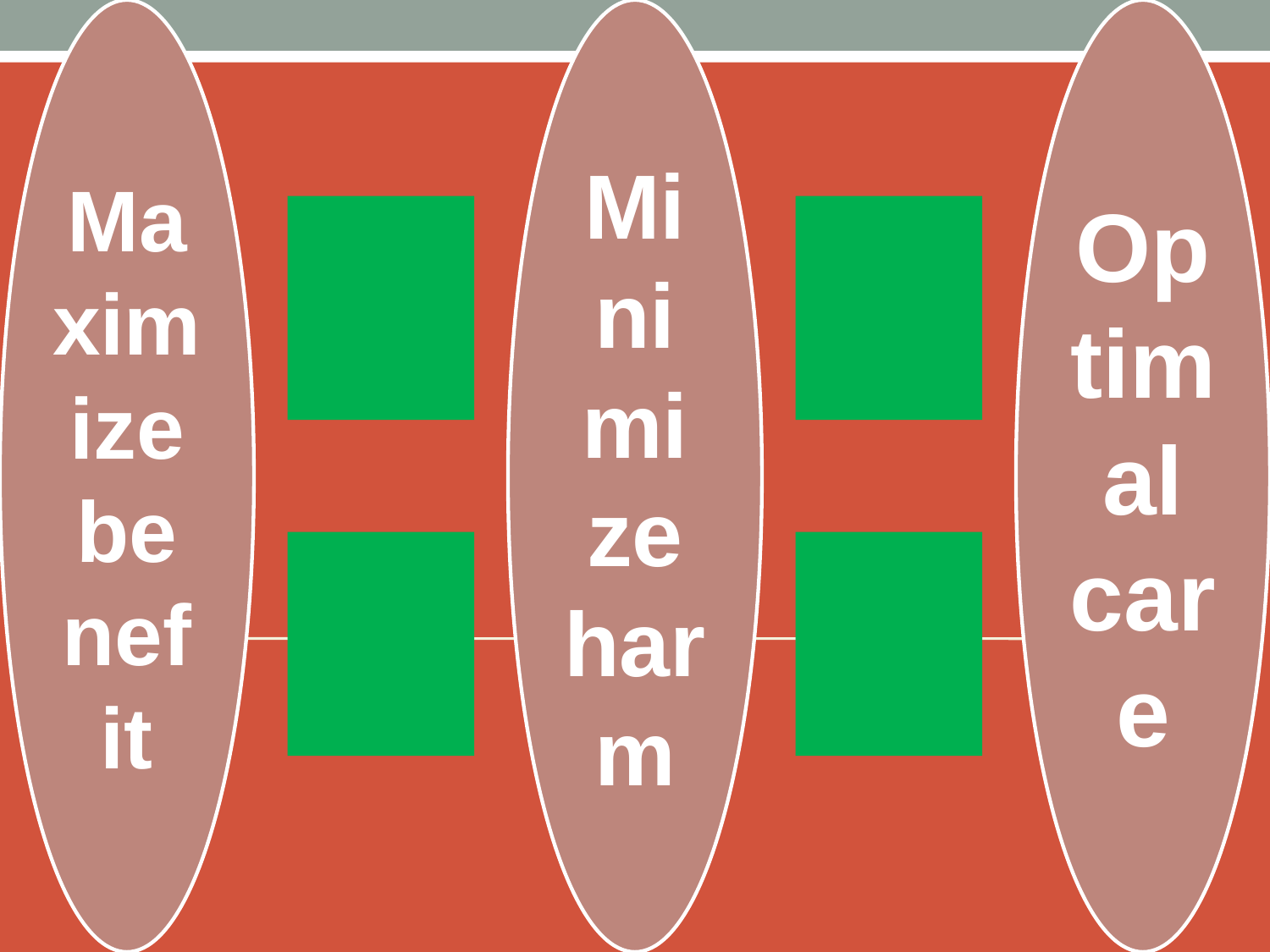

## Slide 16
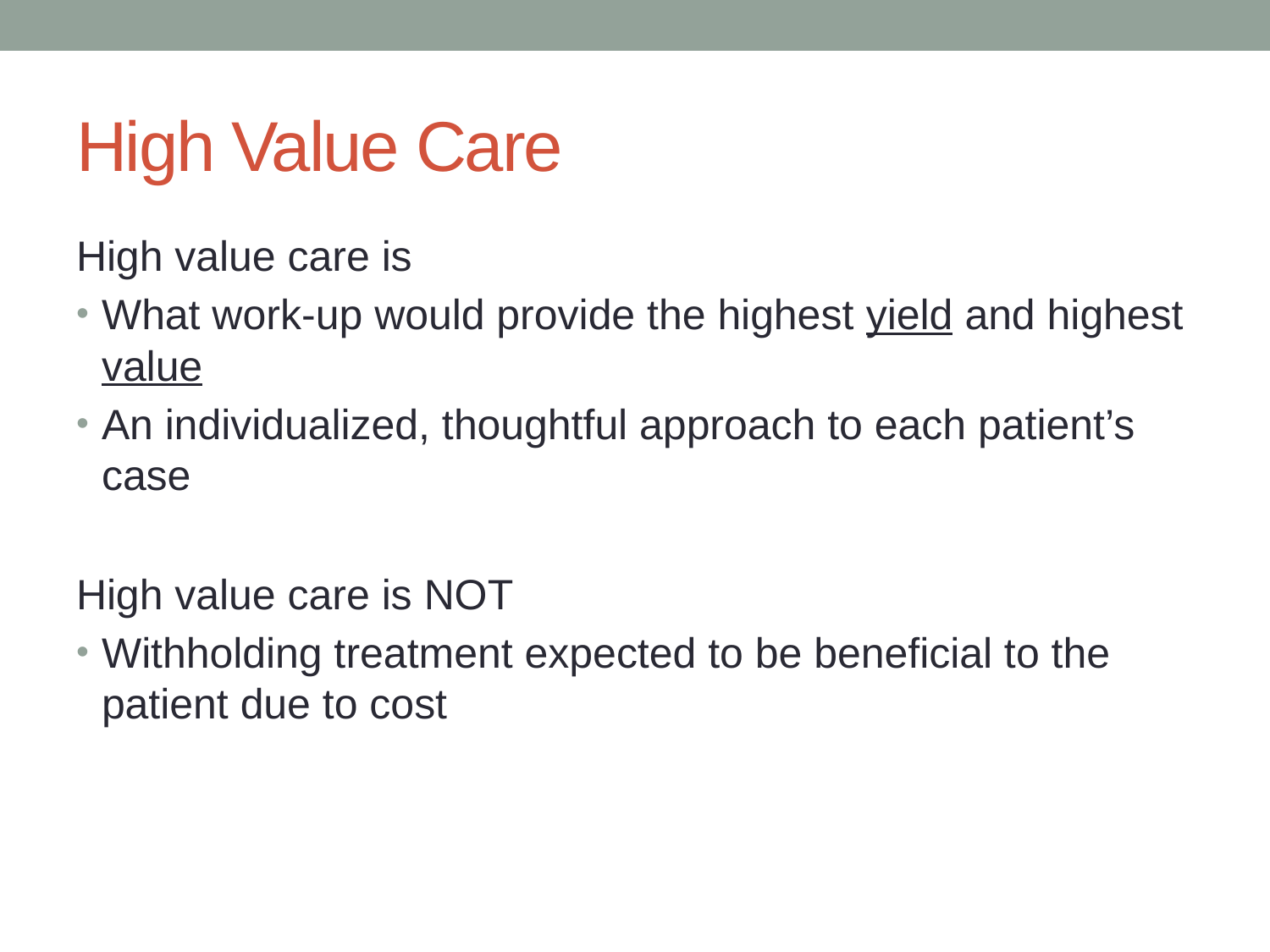

# High Value Care
High value care is
What work-up would provide the highest yield and highest value
An individualized, thoughtful approach to each patient’s case
High value care is NOT
Withholding treatment expected to be beneficial to the patient due to cost

## Slide 17
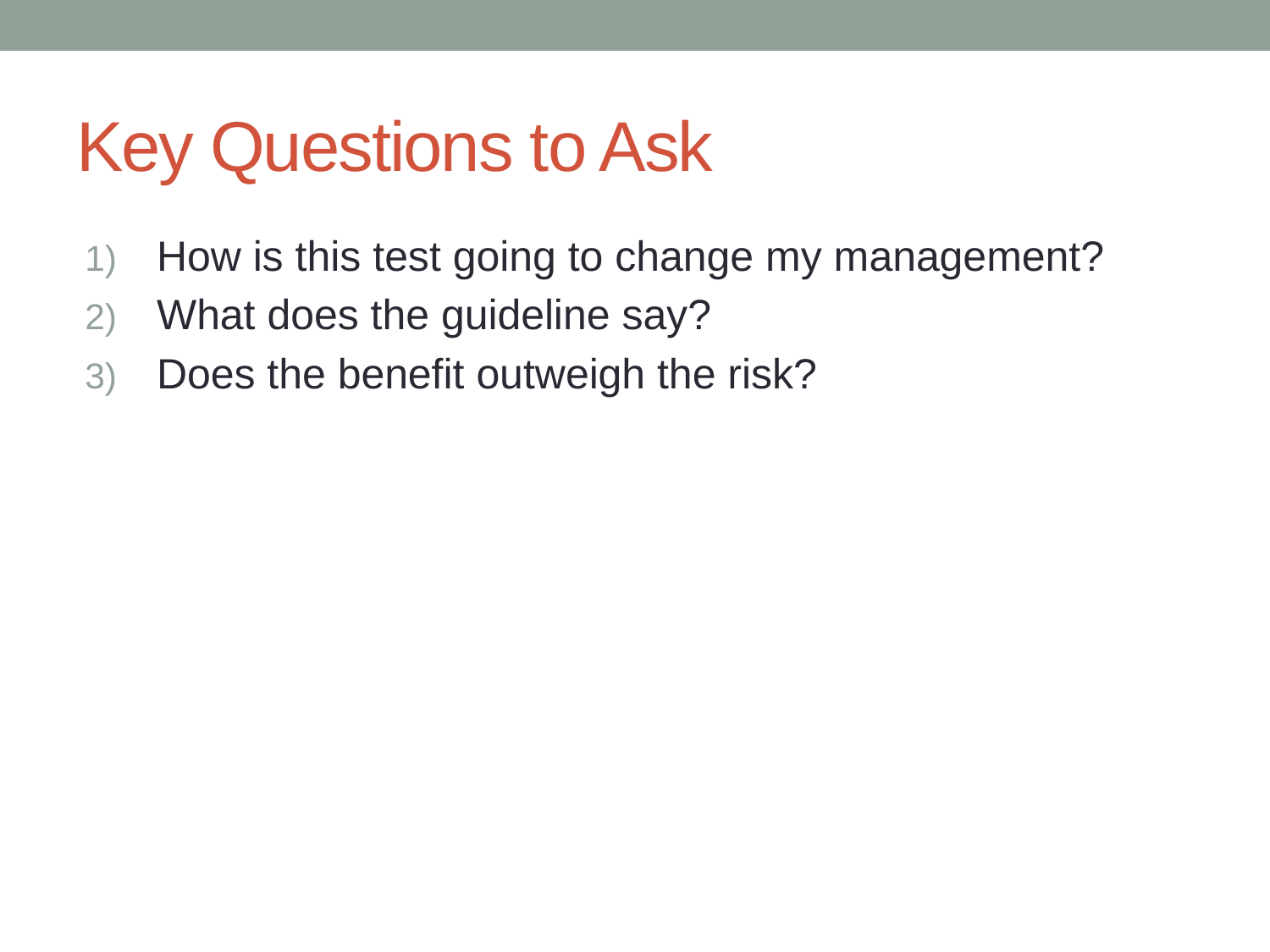

# Key Questions to Ask
How is this test going to change my management?
What does the guideline say?
Does the benefit outweigh the risk?

## Slide 18
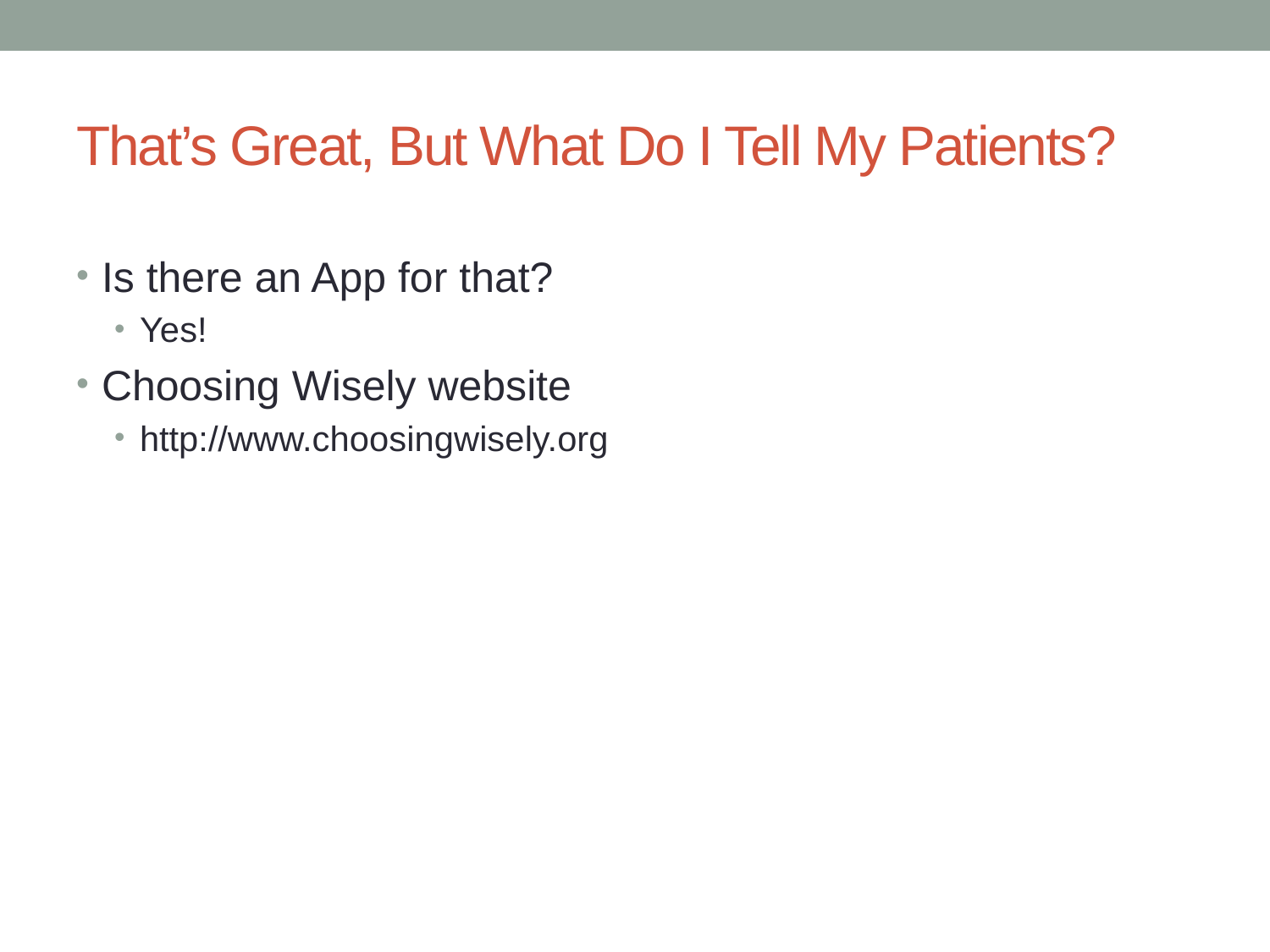

# That’s Great, But What Do I Tell My Patients?
Is there an App for that?
Yes!
Choosing Wisely website
http://www.choosingwisely.org

## Slide 19
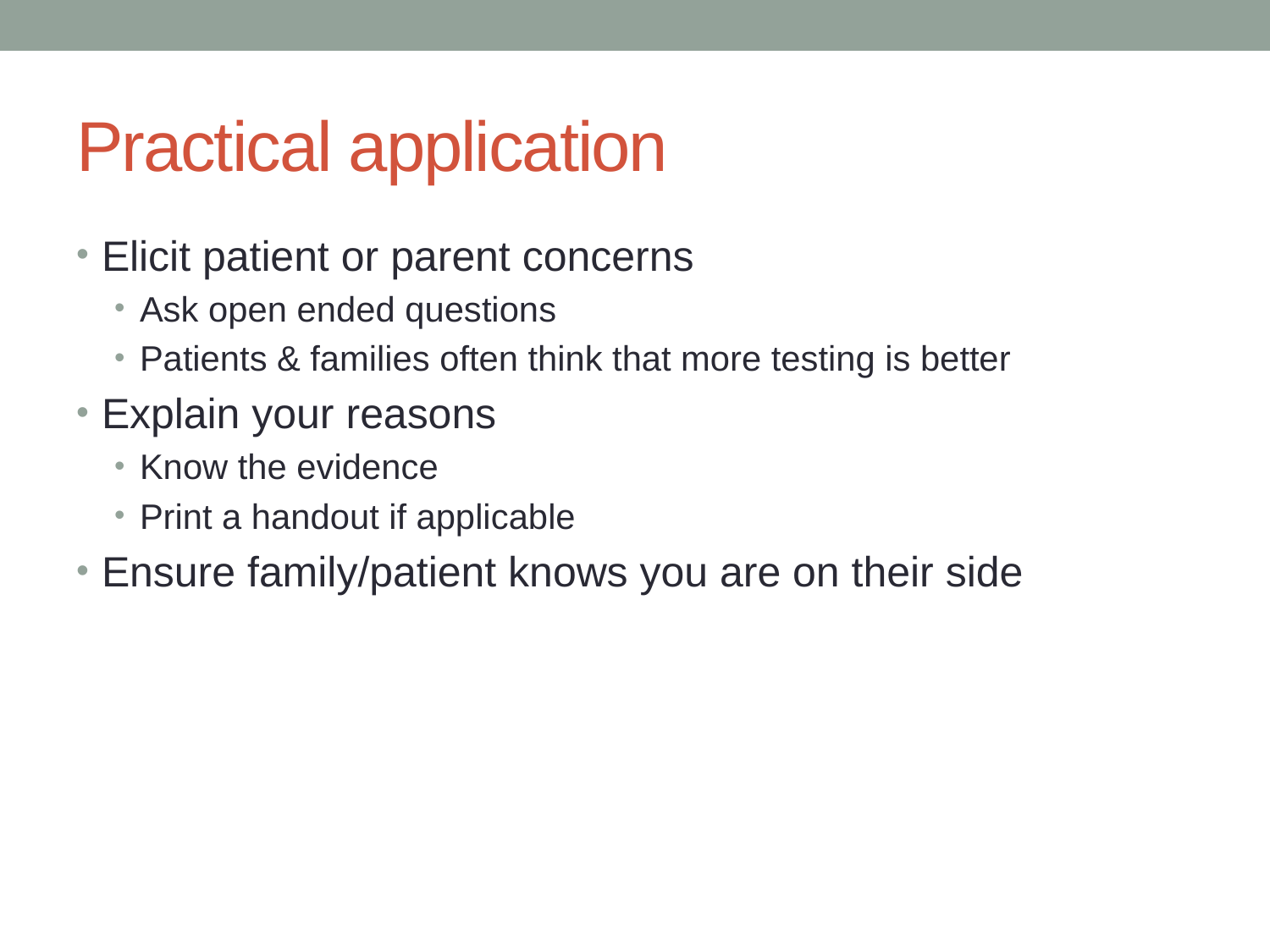

# Practical application
Elicit patient or parent concerns
Ask open ended questions
Patients & families often think that more testing is better
Explain your reasons
Know the evidence
Print a handout if applicable
Ensure family/patient knows you are on their side

## Slide 20
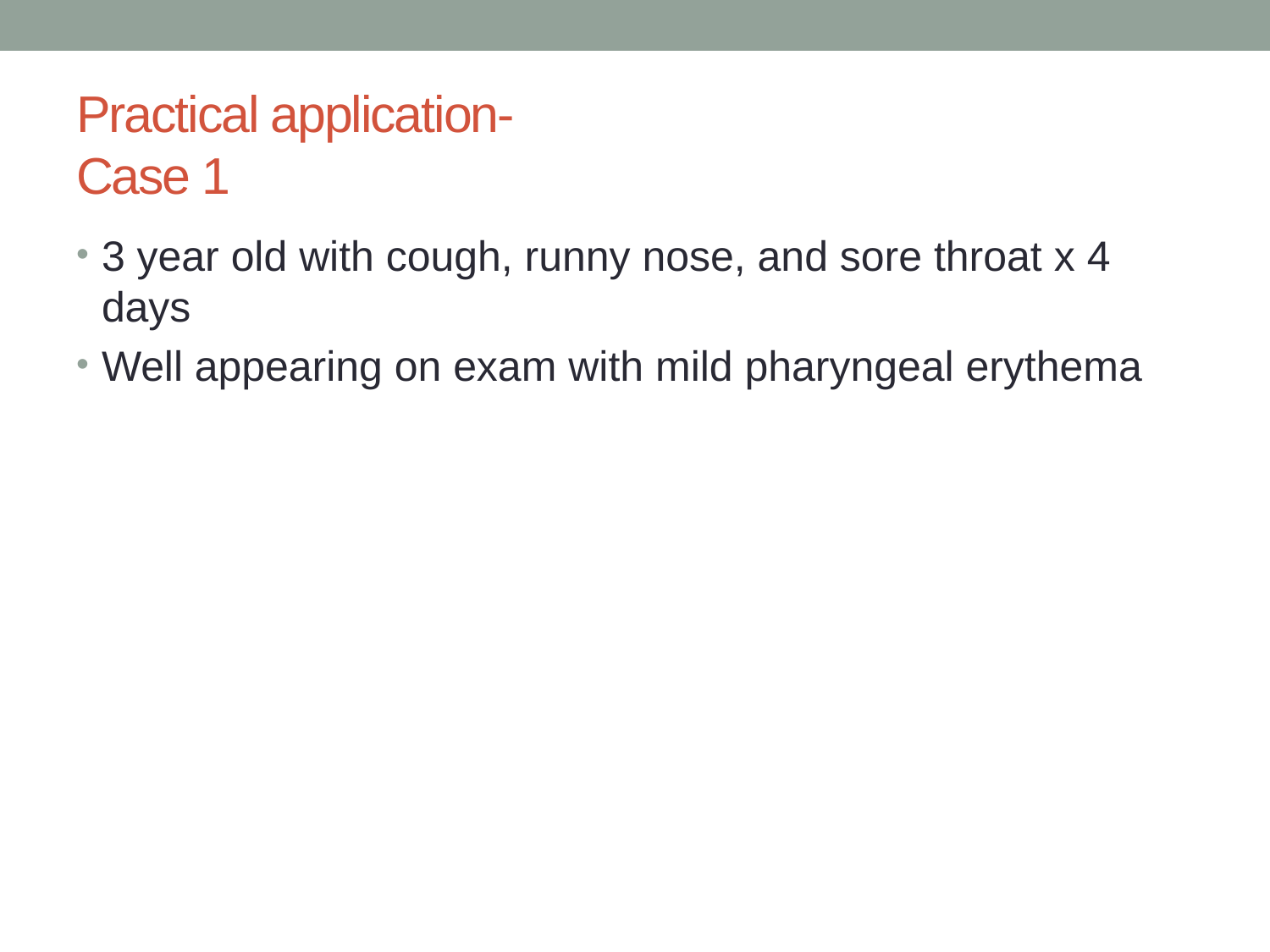

# Practical application- Case 1
3 year old with cough, runny nose, and sore throat x 4 days
Well appearing on exam with mild pharyngeal erythema

## Slide 21
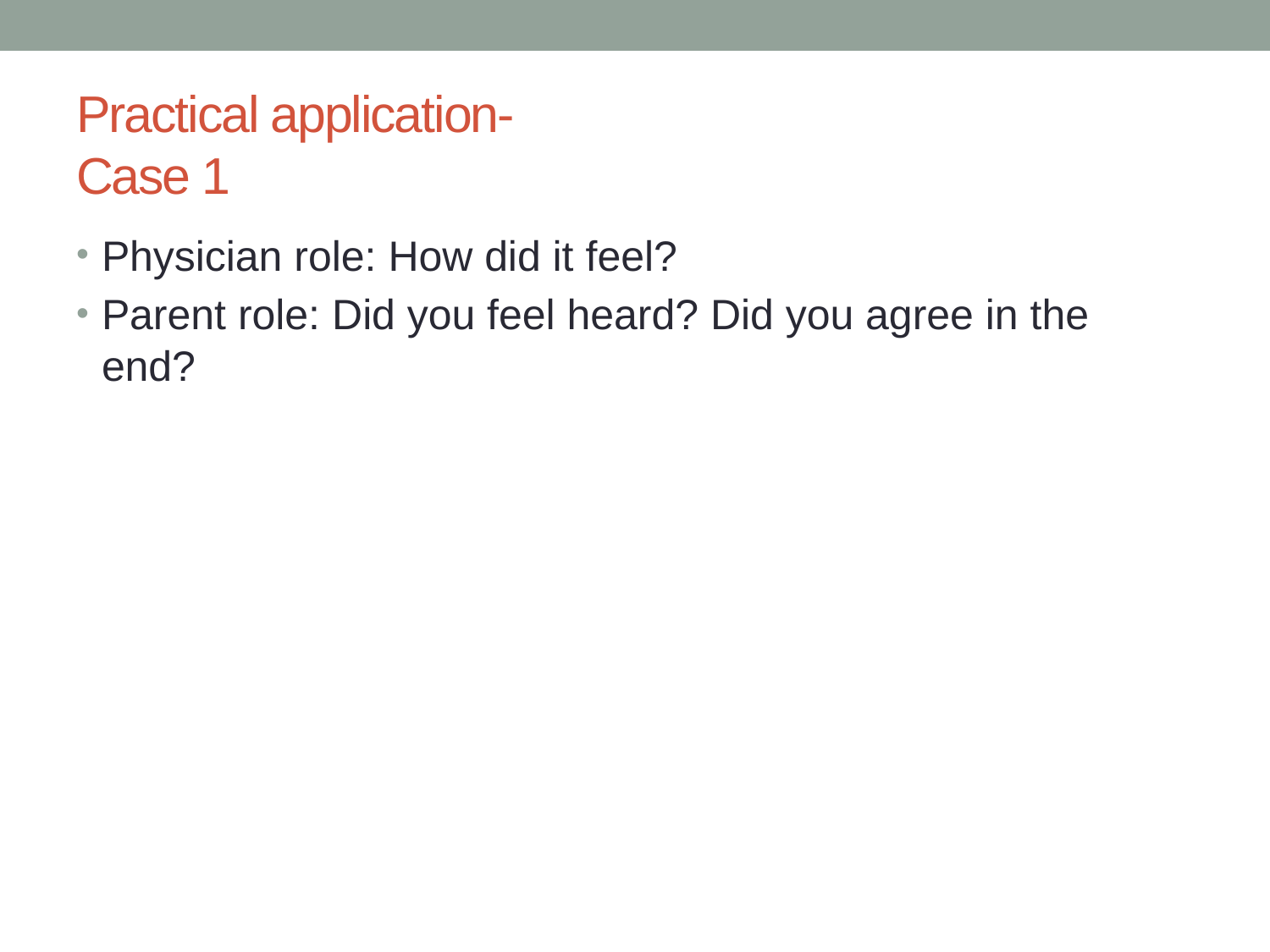

# Practical application- Case 1
Physician role: How did it feel?
Parent role: Did you feel heard? Did you agree in the end?

## Slide 22
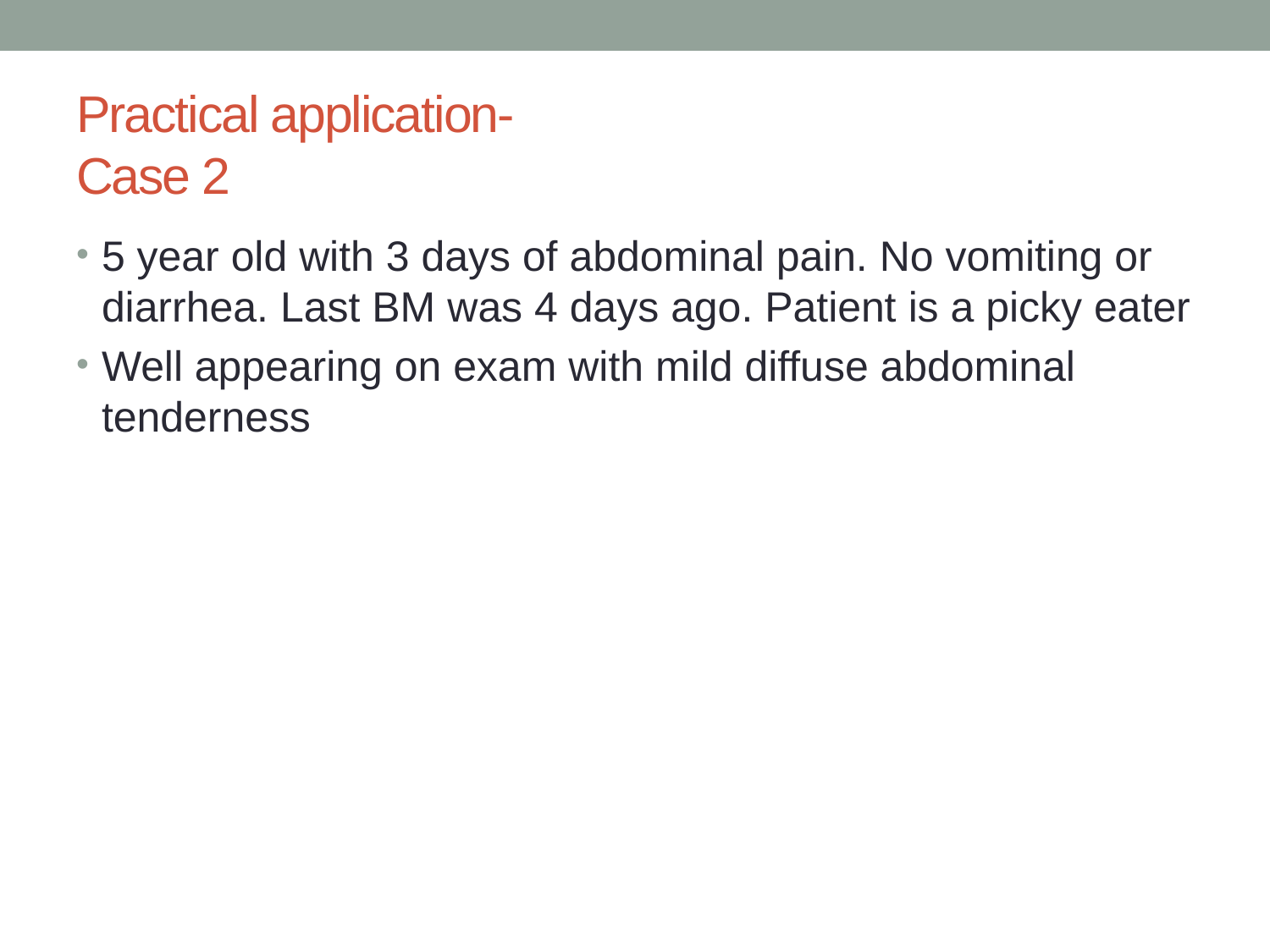

# Practical application- Case 2
5 year old with 3 days of abdominal pain. No vomiting or diarrhea. Last BM was 4 days ago. Patient is a picky eater
Well appearing on exam with mild diffuse abdominal tenderness

## Slide 23
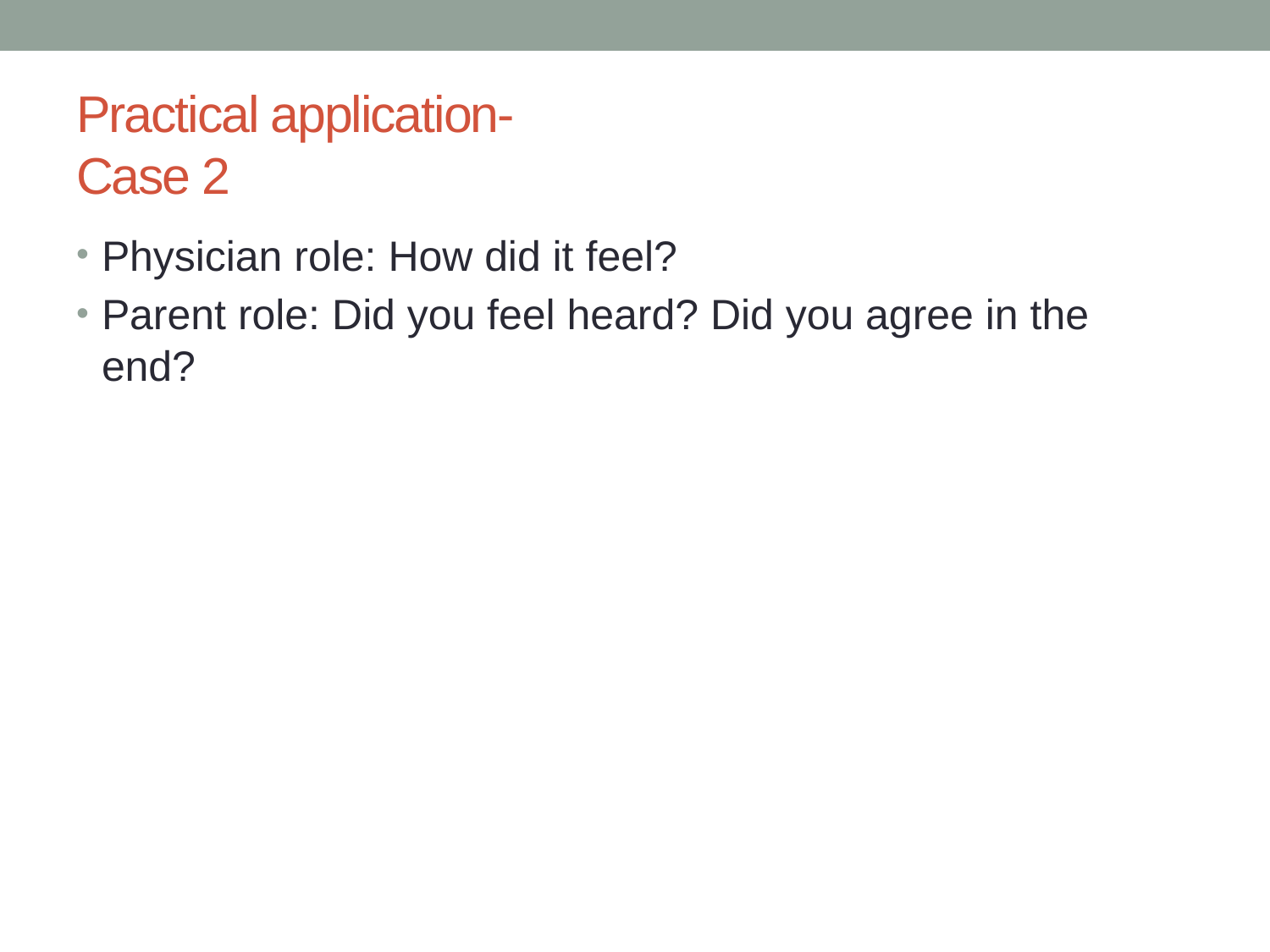

# Practical application- Case 2
Physician role: How did it feel?
Parent role: Did you feel heard? Did you agree in the end?

## Slide 24
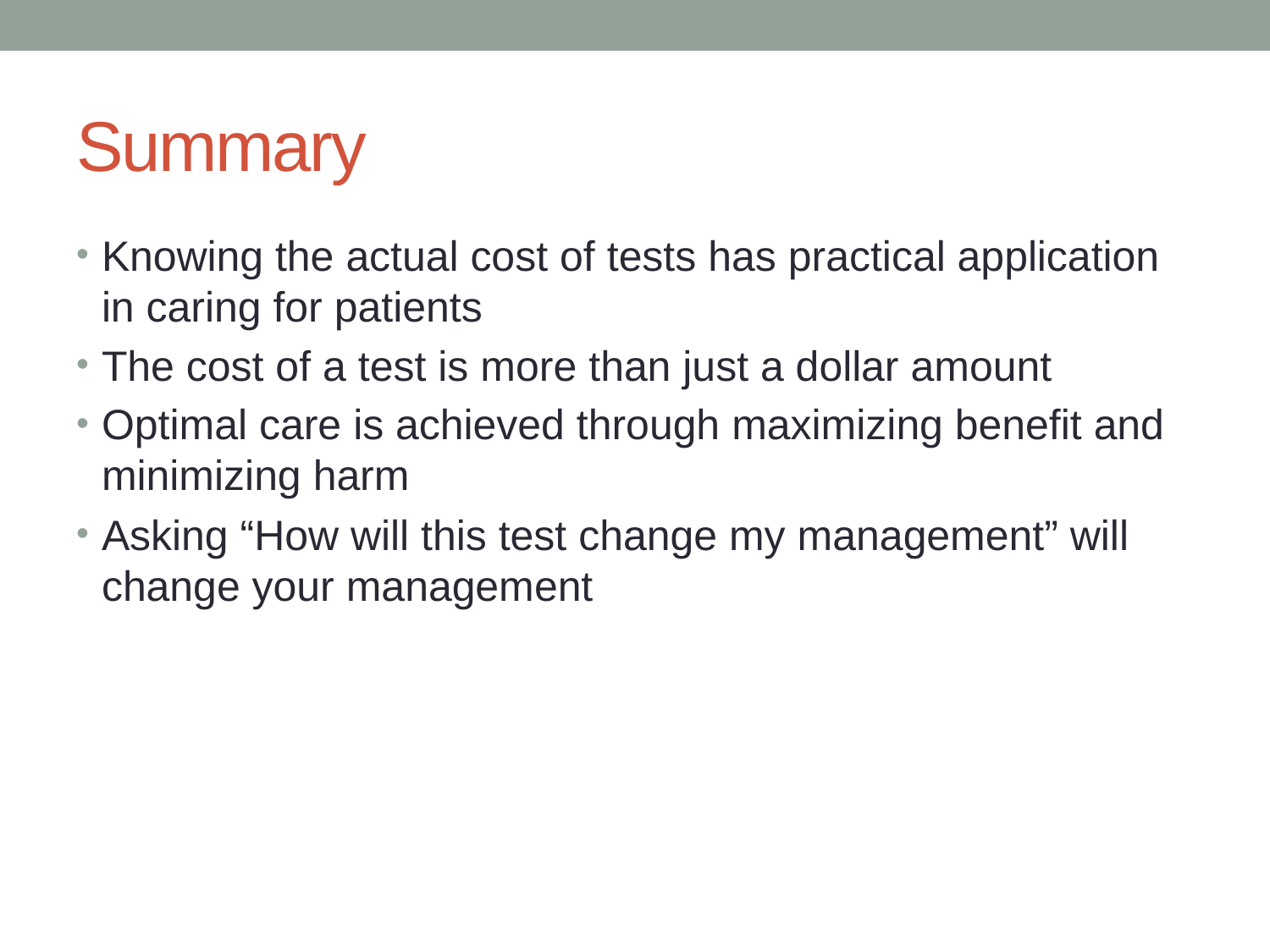

# Summary
Knowing the actual cost of tests has practical application in caring for patients
The cost of a test is more than just a dollar amount
Optimal care is achieved through maximizing benefit and minimizing harm
Asking “How will this test change my management” will change your management

## Slide 25
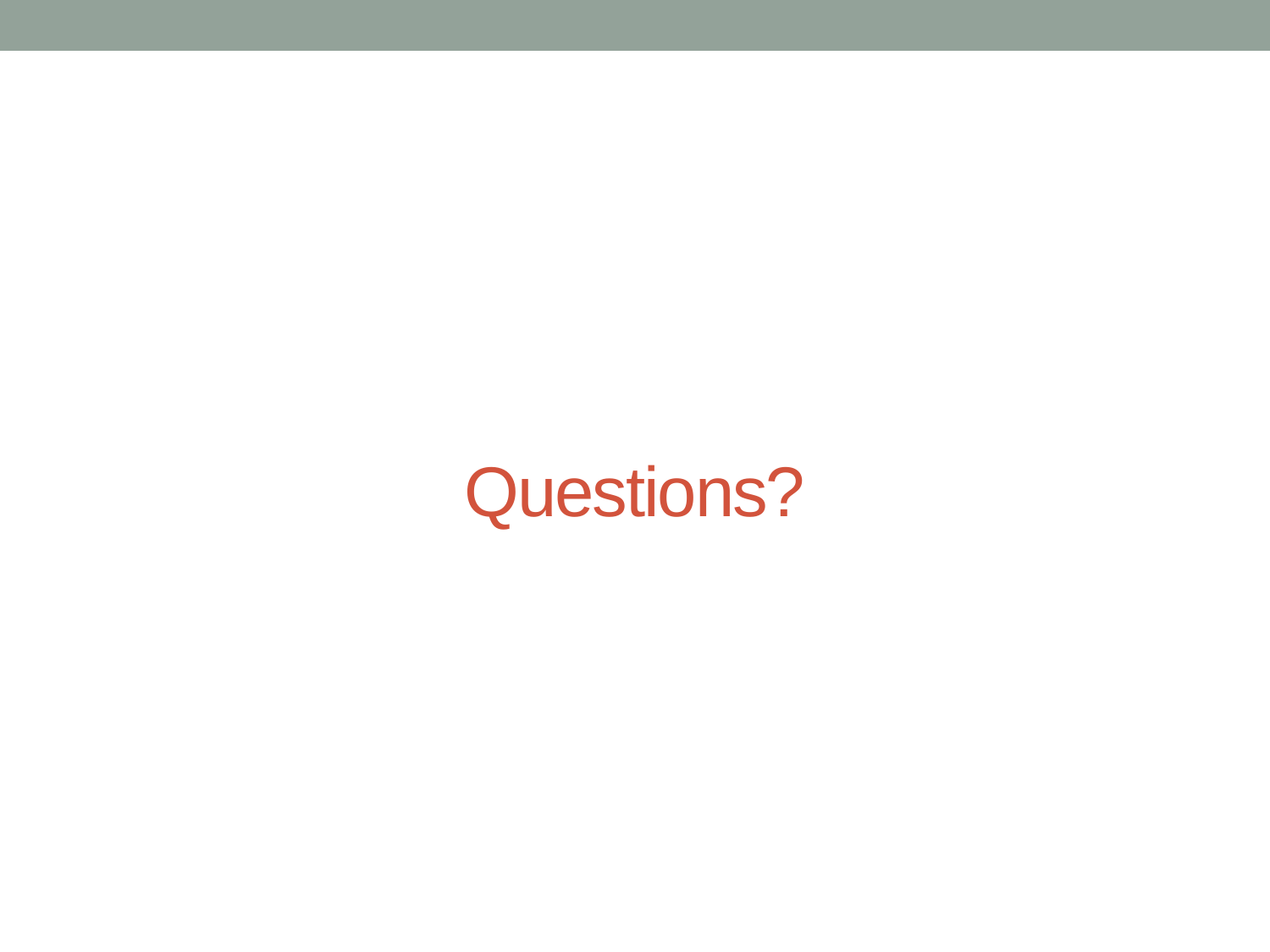

# Questions?
