# Supplementary material for: Applying High-Value Care Principles in a Pediatric Case: A Workshop for Health Professions Students
Source: MedEdPORTAL. 2020 Nov 17;16:11030. doi: 10.15766/mep_2374-8265.11030 (PMC7678025; doi:10.15766/mep_2374-8265.11030)
Supplement: Supplementary file 1 — Facilitator Guide.docxClinical Vignette.docxPowerPoint Presentation.pptxCost List.xlsxRole-Play Cases.docxPre- and Postsurvey.docx [file mep_2374-8265.11030-s001.zip › E. Role-Play Cases.docx]

Role Play Cases- Appendix E

Directions: The following cases are to be used in 2 role plays. Students should pair up. For case 1, one student plays the parent and the other plays the physician. Students should switch roles for case 2.

STUDENT 1:

Case 1- As a parent, you want to give your child cough medicine.

You are worried that the cough is harmful to your child, keeping her up at night, and want to make the cough stop.

Your other concerns are that the child had a fever to 102, 3 days ago. No more fevers. And that she has a runny nose.

Your biggest concern is that you are planning to go on vacation in a few days and what if your child gets worse, since she’s already been sick for 4 days.

Case 2- Physician Tip Sheet on imaging in abdominal pain

- Most abdominal pain in children is caused by constipation.
- Risks of imaging:
  - CT scans use radiation, which can increase the lifetime risk of cancer.
    - The increased lifetime risk for cancer due to excess radiation exposure is of special concern given the acute sensitivity of children’s organs.
    - There also is the potential for radiation overdose with inappropriate CT protocols.
  - Unnecessary CT scans can lead to more tests and treatments, with more risks.
- CT scans are expensive, and an unnecessary cost if not indicated.

STUDENT 2:

Case 1- Physician Tip Sheet on cough medicine

- Over-the-counter cough and cold medicine is not recommended for kids 4 and under.
- Research has shown these products offer little benefit to young children and can have potentially serious side effects. Many cough and cold products for children have more than one ingredient, increasing the chance of accidental overdose if combined with another product.
- Most of the time symptoms are caused by a virus. Medications cannot cure viruses. Symptoms usually resolve in 4-5 days.
- If your child has a stuffy or runny nose, the mucus running down the throat could be causing some of the coughing. A saline nasal spray and frequent nose blowing (instead of sniffling) can help.
- Cost- Most cough syrups do not cost a lot. But money spent on medicines that are not needed is money wasted.

Case 2- As a parent, you want your child to get a CT scan to figure out what’s wrong

You have a friend whose child had appendicitis and so you want to make sure nothing is missed.

Your child has had this kind of pain before so how can you make sure it doesn’t come back.
