# Supplementary material for: Applying High-Value Care Principles in a Pediatric Case: A Workshop for Health Professions Students
Source: MedEdPORTAL. 2020 Nov 17;16:11030. doi: 10.15766/mep_2374-8265.11030 (PMC7678025; doi:10.15766/mep_2374-8265.11030)
Supplement: Supplementary file 1 — Facilitator Guide.docxClinical Vignette.docxPowerPoint Presentation.pptxCost List.xlsxRole-Play Cases.docxPre- and Postsurvey.docx [file mep_2374-8265.11030-s001.zip › F. Pre- and Postsurvey.docx]

**Appendix F**

**Pre-Intervention Survey**

**Unique ID** (first 2 letters of parent’s name + 2-digit birth month): **_______**

**Please answer all questions as you would have prior to taking this session.**

1. What exposure have you had to education on cost-effective medical care prior to today?
   1. None
   2. Some
   3. A lot
2. When making medical decisions in the inpatient setting, how often did you factor in the cost of tests/imaging/medications prior to today’s session?
   1. Never
   2. Sometimes
   3. Usually
   4. Always
3. How would you rate your understanding of the actual cost to the hospital for patient care prior to today’s session?
   1. Completely unaware
   2. Minimally knowledgeable
   3. Moderately knowledgeable
   4. Expert
4. How important did you think incorporating cost-effectiveness into your patient care was prior to today’s session?
   1. Not important
   2. Somewhat important
   3. Very important
5. What motivated you to think about cost as it pertains to patient care prior to today’s session (choose all that apply)?
   1. Cost to my patients
   2. Cost to the hospital
   3. Cost to the overall U.S. healthcare system
   4. I didn’t think about cost
   5. Other: _____________________________________

**Post-Intervention Survey**

**Unique ID** (first 2 letters of parent’s name + 2-digit birth month): **_______**

1. This session was helpful for me:
   1. Yes
   2. No
   3. Other: _____________________________
2. Has your knowledge of high-value, cost-conscious patient care improved after this session?
   1. No, not at all
   2. Only slightly improved
   3. Yes, moderately improved
   4. Yes, significantly improved
3. After this session, when making medical decisions in the inpatient setting, how often do you think you will factor in the cost of tests/imaging/medications?
   1. Never
   2. Sometimes
   3. Usually
   4. Always
4. After this session, how would you rate your understanding of the actual cost to the hospital for patient care?
   1. Completely unaware
   2. Minimally knowledgeable
   3. Moderately knowledgeable
   4. Expert
5. After this session, how important do you think incorporating cost-effectiveness into your patient care is?
   1. Not important
   2. Somewhat important
   3. Very important
6. After this session, I will change my practice to reflect more cost-conscious care:
   1. Yes
   2. No
   3. Other: _____________________________
7. If you answered yes to question 6, please list the ways you think your practice will change
