# Supplementary material for: Applying High-Value Care Principles in a Pediatric Case: A Workshop for Health Professions Students
Source: MedEdPORTAL. 2020 Nov 17;16:11030. doi: 10.15766/mep_2374-8265.11030 (PMC7678025; doi:10.15766/mep_2374-8265.11030)
Supplement: Supplementary file 1 — Facilitator Guide.docxClinical Vignette.docxPowerPoint Presentation.pptxCost List.xlsxRole-Play Cases.docxPre- and Postsurvey.docx [file mep_2374-8265.11030-s001.zip › _Educational_Summary_Report_11030.pdf]

MedEdPORTAL® The AAMC Journal of Teaching and Learning Resources

Original Publication Open Access

Applying High-Value Care Principles in a Pediatric Case: A Workshop for Health Professions Students

Priya N. Jain, MD*, Steven Eagle, MD, Miriam Schechter, MD, Hai Jung H. Rhim, MD, Rhonda Acholonu, MD

*Corresponding Author: priya.j.narayanan@gmail.com

Abstract

Introduction: The cost of health care in the US is rapidly rising. Understanding the financial cost of medical care is an important competency for physicians and physicians-in-training. Medical students in their clinical clerkships are being exposed to health care decision-making often for the first time and are forming habits they will carry throughout their training and careers. Teaching high-value care (HVC) principles is crucial for students as they will be the future leaders in health care. **Methods:** This 1-hour workshop was interactive and aimed to prepare medical students to apply HVC when making medical decisions. The topic of HVC was initially introduced by eliciting tests that students want to order and highlighting the concept of whether or not a test changes the management of the patient. This exercise was followed by a PowerPoint presentation which discussed HVC, Choosing Wisely guidelines in pediatrics, and how to communicate with parents and patients about this topic. **Results:** Of third-year medical students, 125 participated in the workshop, with a survey response rate of 90% ($n = 112$). Ninety-nine percent reported that this workshop was helpful, and 97% reported that they will change their practice to reflect more cost-conscious care. Most students reported that their knowledge of HVC improved after the session, with 88% reporting scores of 3 (*moderately improved*) or 4 (*significantly improved*). **Discussion:** This ready-to-implement workshop offered students an understanding of how the cost of medical care impacts patients and families and how to practice cost-conscious care in pediatrics.

Keywords
Pediatrics, High-Value Care, Case-Based Learning, High-Value Care/Cost-Conscious Care, Internal Medicine, Physician

Educational Objectives

By the end of this activity, learners will be able to:

1. Describe high-value cost-conscious care.
2. Analyze cost to the national health care system, as well as cost to the patient, when making diagnostic and therapeutic decisions in order to decrease unnecessary health care spending.
3. Examine harms and non-monetary costs when making medical decisions.
4. Develop strategies to communicate with patients and families in navigating high-value care discussions.

Citation:
Jain PN, Eagle S, Schechter M, Rhim HJH, Acholonu R. Applying high-value care principles in a pediatric case: a workshop for health professions students. *MedEdPORTAL*. 2020;16:11030.
https://doi.org/10.15766/mep_2374-8265.11030

Introduction

Studies project that by 2025, nearly 20% of the United States gross domestic product will consist of health care expenditures.[1] Additionally, according to a National Academy of Medicine report, over ∃750 billion of total US health care spending was potentially avoidable.[2,3] The report also stated that national health spending growth is projected to outpace projected growth in gross domestic product.[2] With rapidly rising costs of health care placing a strain on the US health care delivery system, understanding the financial cost of medical care is an important competency for physicians and physicians-in-training. In order to provide high-value care (HVC) when making clinical decisions, physicians are not only expected to use their clinical acumen to identify appropriate diagnostic testing and treatment options, but are also tasked with balancing potential benefits with the harms and financial burden of treatment.[4]

Understanding how the cost of medical care impacts patients and their families is important because upfront costs to patients are some of the most well understood social determinants of

Copyright © 2020 Jain et al. This is an open-access publication distributed under the terms of the Creative Commons Attribution license.

health. Perceived cost of care is a greater barrier to persons with low incomes, unemployed, and uninsured patients.[5] In a cross-sectional survey study of adult oncology patients, 39% of patients surveyed reported altering their care (e.g., not filling a prescription) and 89% their lifestyle (e.g., spending less on food and clothing) due to treatment-related financial distresss.[6] While cost-conscious health care spending requires a comprehensive interdisciplinary approach, the physician plays a critical role in implementing high-value medical decision-making that decreases unnecessary and expensive care for patients and the US health care system. Physicians who understand cost to the health care system and the patient are better equipped to provide HVC that is individualized, patient-centered, and effective.

Teaching high-value and cost-conscious care to physicians-in-training is not only necessary but is also highly desired.[7-11] The Choosing Wisely campaign, for example, helps physicians to select tests and procedures that are evidence-based, free of harm, not duplicative, and truly necessary.[12,13] Educational interventions on HVC have been successful at increasing physician awareness of medical costs.[14-18] It has been suggested, in fact, that physician education regarding cost-conscious care and overuse or misuse of diagnostic testing become an additional ACGME general competency for physicians-in-training.[9,19]

Studies have shown that physicians and trainees have a poor understanding of pharmaceutical and diagnostic testing costs.[20] A survey of pediatric residents and attending physicians at the Children's Hospital of Philadelphia in 2011 revealed that a majority of both residents and attendings feel "minimally knowledgeable" or "completely unaware" of hospital finances.[21] Moreover, only 15% of attendings and 11% of residents were able to accurately estimate costs of common tests, medications, and services.[21] This may, in part, be due to the fact that cost-effectiveness is not often taught as part of medical school and residency curricula.[7,8,22] Medical students in their clinical clerkships are being exposed to health care decision-making often for the first time and are forming habits they will carry throughout their training and careers. For this reason, teaching HVC principles is crucial for students as they will be the future drivers in health care. While a few studies have implemented curricula for residents[23-27] and medical students in internal medicine,[28,29] there are no studies to our knowledge, including on *MedEdPORTAL*, examining the effect of a pediatric-based curriculum on medical students alone. Unlike existing workshops,[23,29] our session was delivered in a single 1-hour session, making it efficient and easily incorporated into an existing conference schedule.

In order to address this gap, we developed a case-based, interactive workshop designed to increase medical students' knowledge of high-value medical care and the impact of overuse or misuse of diagnostic testing across the domains of cost to the US health care system and cost to the patient, including harm, and applied these concepts in pediatric practice. Our workshop was unique in that learners were not told the session was about HVC, and so the topic of value-added care was initially introduced by eliciting tests that students wanted to order and highlighting the concept of whether or not a test changes the management of the patient. This allowed students to make connections between new information and their current thinking, and potentially confront misconceptions.[30] This session also utilized different active and collaborative learning strategies[31-33] including small-group team-based learning, case-based discussion, and role-play in order to reinforce the concepts of HVC, making it different from the existing literature. This resource was intended to be ready to implement.

Methods

Learners and Facilitators

Learners were all Albert Einstein College of Medicine students on their pediatrics clerkship. At the time of the session, students had spent some time in the inpatient or outpatient setting, or in some cases both. There were four facilitators, with some knowledge of HVC, who each administered the session (one facilitator per session). Facilitators met for 3 hours prior to the session for review and training. Training sessions entailed reviewing the teaching materials; discussion of the potential differential diagnosis and diagnostic plans that students could generate; how best to ask the impact of each test; and time management of the session. The facilitator guide (Appendix A) was utilized for all facilitators. Facilitators were pediatric faculty. Prerequisite knowledge of the basic concepts of HVC was helpful but not essential to facilitating these sessions.

Curriculum Overview

This curriculum was created by two pediatric hospital medicine faculty and approved for implementation by the pediatric clerkship leadership. The curriculum consisted of a 1-hour small-group session for students on their pediatric clerkship; the outline and timeline of the session are available in the facilitator guide. Each session consisted of approximately 10 students. There was no required preassignment. The session began with a clinical vignette (Appendix B) that students discussed in small groups, formulating a differential diagnosis and initial diagnostic and management plan. Students were not initially told that the session was about HVC. The differential and management plan

Copyright © 2020 Jain et al. This is an open-access publication distributed under the terms of the Creative Commons Attribution license.

from each team was then discussed in the larger group. The facilitator asked how each diagnostic test suggested impacted clinical decision-making. After the case was discussed, the true intention of HVC was revealed. The session continued with a didactic portion (Appendix C) including a discussion of the cost of tests and treatments (Appendix D as a reference). This was followed by a role-play experience where the students alternated playing a parent or a physician in two case scenarios (Appendix E) discussing unnecessary interventions or testing. At the end of the session, all participants were asked to anonymously and voluntarily complete a retrospective pre/postsurvey (Appendix F). The study was approved by the Albert Einstein College of Medicine Institutional Review Board.

Equipment

- Computer with linked screen for projection of PowerPoint presentation (suggested).
- Whiteboard or flip chart and colored markers for recording group work (suggested).
- Timer (recommended but not required).

Outline for the 1-Hour Workshop

- Case presentation with small-group discussion (10 minutes).
- Large-group debrief (10 minutes).
- PowerPoint introduction of Choosing Wisely Campaign and HVC principles (20 minutes).
- Practice role-playing with debrief (10 minutes).
- Summary and self-evaluation (10 minutes).

Case Presentation With Small-Group Discussion

Students were asked to break in to groups of three or four to discuss the vignette of a 14-month-old afebrile male with respiratory distress, copious nasal secretions, with coarse breath sounds, but without wheezing. Students generated a list of differential diagnoses, initial diagnostic, and management plan. The vignette was developed with the intended diagnosis of bronchiolitis, but allowed for the suggestion of alternate diagnoses, such as reactive airway disease or pneumonia. Because students were not initially told that the topic of the session was HVC, we suggested an alternate title for the presentation, such as "Management of the Pediatric Patient," or another option that does not allude to HVC. The suggested time for this initial activity is no more than 10 minutes.

Large-Group Debrief

Each small group provided their top three differential diagnoses, written on the whiteboard in a different color per group. After each group shared their top three, the facilitator opened the discussion to all groups to add diagnoses until all possibilities had been shared. The facilitator did not give feedback on the accuracy of differential diagnoses at this point. The facilitator then prompted each group to share an initial diagnostic plan (ideally in reverse order of differential sharing). The facilitator asked, "What does this help you rule in or rule out?" after each test was requested. For example, if a group said a chest X-ray to rule out pneumonia, the facilitator drew an arrow to pneumonia on the differential. If pneumonia was not on the original list of differential diagnoses, the facilitator pointed this out and asked if pneumonia needed to be added. The facilitator drew arrows to as many diagnoses on the list as applicable. The facilitator also started to introduce some ideas of how tests may not change management. For example, if a complete blood count was requested, the facilitator asked if a normal white blood cell count (WBC) was possible in bacterial infection or if WBC can be elevated in viral illness. The facilitator asked if any additional tests were needed (i.e., blood or urine culture).

Didactic Slide Presentation

This resource (Appendix C) was a PowerPoint-based interactive workshop discussing HVC, Choosing Wisely guidelines in pediatrics, and how to communicate with parents and patients about this topic. The Choosing Wisely campaign, initially an initiative of the American Board of Internal Medicine Foundation which has been adopted by 50 specialty societies, was used as the basis of this workshop.[12] The principles of this campaign as well as specific recommendations within pediatrics were introduced to learners in order to encourage them to think and talk about medical tests and procedures that may be unnecessary and that in some instances can cause harm. When discussing the cost of health care, we distributed the cost sheet (Appendix D) as a reference. The cost sheet was developed from the Healthcare Bluebook website.[34] Reported prices were Bluebook's fair prices and were based on the actual amount paid on the claim, not the billed amount.

Practice Role-Playing With Debrief

Learners participated in one or two role-playing cases. Working in dyads, one was the parent and the other was the health care professional. Case 1 discussed the need for cough syrup or antibiotics in a child with upper respiratory infection symptoms; case 2 discussed the need for abdominal imaging in a child with abdominal pain likely due to constipation. The group came together to debrief as a whole after case 1 with the facilitator prompting for difficult encounters and suggestions for future encounters. The parent role also expressed if they felt their concerns were appropriately addressed. If time permitted,

Copyright © 2020 Jain et al. This is an open-access publication distributed under the terms of the Creative Commons Attribution license.

learners switched roles and role-played the second case provided, followed by a group debrief again.

Summary and Self-Evaluation

Learners had the option to complete a brief deidentified retrospective pre/postsurvey directly after the intervention session, rating their understanding of HVC and whether the workshop would impact future decision making. The retrospective pre/post design was used in order to keep the intention of HVC from being revealed at the start of the session, and to mitigate response shift bias.[35-37] A written presurvey consisting of six questions and postsurvey consisting of eight questions were developed by the study investigators. The following domains were addressed in the presurvey: attitudes towards HVC and baseline knowledge of the cost of patient care to the hospital. The following domains were addressed in the postsurvey: attitudes towards HVC, understanding of cost to the hospital, as well as curriculum feedback. Pre- and postintervention surveys were tested for readability and understanding prior to full implementation.

Data were analyzed with paired *t* tests using Microsoft Excel. For continuous data, mean was used as a measure of central tendency. Range and standard deviation were used as measures of dispersion. Standard error of mean and 95% confidence intervals were calculated to estimate the true populations mean for each group. Noncontinuous data was reported in frequencies and percentages/proportions. Responses to Likert-style questions were treated as ordinal data with central tendencies summarized by median. Qualitative data were analyzed for themes. Analysis started with a free reading of the written comments by two independent researchers. During a second reading of the text, the two researchers independently performed categorization of emerging themes. Each researcher's results were reviewed by the entire research team.

Results

This workshop has been implemented at the Albert Einstein College of Medicine for over 2 years for students on their pediatrics clerkship. During the data collection phase, there were 125 participants in this workshop, with 112 who completed both the pre-and postsurvey (90% completion rate). Most students (83%, $n = 93$) reported some education on cost-effective medical care prior to this workshop ($n = 7$ reported *none*, $n = 12$ reported *a lot*). Of students, 110 of 112 (99%) reported that this workshop was helpful and 109 (97%) reported that they will change their practice to reflect more cost-conscious care. Using a 4-point Likert scale, most students reported that their knowledge of HVC improved after the session, with 88% reporting scores of 3 (*moderately improved*) or 4 (*significantly improved*). The mean of responses reporting understanding of cost to the hospital rose significantly (pre = 2.2 (0.53), post = 2.7 (0.46), $p < .001$) after the intervention.

In addition to the Likert-style questions, one open-ended question at the end of the survey asked participants to identify any changes in behavior as a result of the session. A thematic analysis of qualitative responses was performed by two reviewers. This analysis revealed student perceptions of the impact of the curriculum on their behavior and future practice. Three major themes emerged from the analysis, as follows: Participants believed that the curriculum will affect their practice through improved utilization of Choosing Wisely, less testing, and more thought/considering downstream effects of medical decisions. Themes with quote examples are presented in the Table.

Discussion

In order to prepare medical students to apply HVC concepts when making medical decisions for pediatric patients, we developed an interactive workshop that reviews concepts of HVC, including the Choosing Wisely lists in pediatrics and encouraged thinking of downstream effects of medical decisions. Overall, this workshop has been well received by participants and similar to other studies,[25-28] participants reported increased confidence and self-assessed competence in providing high-value, cost-conscious care. Students reported that the curriculum improved their knowledge of HVC by pushing them to understand how an intervention changes management and to think about cost and harm. However, unlike previously published interventions,[25-27] our learners were not told that the case discussion was based in HVC concepts which made it more likely that students gave their initial diagnostic plan unbiased

Table. Themes and Example Quotes ($N = 112$)

Theme	Example Quotes
Improved utilization of choosing wisely	"Directly refer to Choosing Wisely when relevant."<br>"Practice/checking Choosing Wisely."
Less testing	"I will make sure the tests I order have significant reason for being ordered and play a role in the clinical course/management of the patient."<br>"Will not order studies if the results will not change management."
More thought/downstream effects	"Blood tests cost money, each hospital day could be a day of missed work."<br>"Think more about downstream effects of ordering tests."<br>"Actively consider cost and necessity when I order diagnostic tests."

Copyright © 2020 Jain et al. This is an open-access publication distributed under the terms of the Creative Commons Attribution license.

by the idea of HVC. Because the facilitator then prompted them to explain why they were asking for a test or intervention and if and how that changed management, this allowed the students to make new connections between their current thinking and HVC. We believed this would solidify these HVC concepts.[30] Students reported that asking "why" could alter the treatment plans of patients as well as shape the learning environment in which they make medical decisions. Additionally, since our respondents were exclusively students on the pediatrics clerkship our free-text responses suggested that students may be able to demonstrate a nuanced understanding of HVC concepts.

This workshop was given at different time points in the clerkship. Students that had some pediatric experience seemed to participate more than if the session was at the beginning of the clerkship. Ideally, this workshop would be given mid-clerkship for maximal benefit of participation as well as of the utilization of the concepts in practice. This session has also recently been given virtually using Zoom and its whiteboard feature. This allowed all students to participate regardless of site of rotation, so could be utilized for remote learning. Breakout rooms were utilized on Zoom in order for students to still have small-group discussions about the initial case.

In reflection, there were some modifications that could be made to our workshop. We used the Healthcare Bluebook as our reference for cost information since it is widely available and standardized.[34] While the Healthcare Bluebook is able to track relative costs, it is based on the actual amount paid on the claim, not the billed amount, so the actual amount charged to the patient may be vastly different. It was important to note that the actual costs borne by the patient could differ significantly based on insurance provider, hospital provider, and even geographic location. Our use of the Healthcare Bluebook was based on zip code, so for future sessions facilitators should modify the costs-based location and local references. One could consider using other sources such as institution-based data or insurance claims data, which may provide cost information that was closer to the actual charge. It would be important to state that charges are different from costs if choosing to use insurance claims data. Facilitators in our sessions were pediatric faculty, but in future sessions, facilitators could be fellows or chief residents. Additionally, we used a respiratory case, but another case could be used to initiate the discussion. Though we gave this workshop to third-year medical students on their pediatrics clerkship, this session could be given during the family medicine clerkship, for fourth-year students, for nurse practitioner/physician assistant students, and possibly even residents. We gave this workshop as an independent session, but it could be utilized in a longitudinal curriculum, aligning it with aspects of HVC in other fields, which could help ensure that these concepts are reinforced over time. While our goal group size per session was 10-12 learners, this session could be done with a larger size group if a spokesperson is nominated from each small group. This was the format we utilized for the virtual session.

Our project had several limitations. Similar to other studies, our biggest limitation was that we did not yet know whether or how this workshop would actually change behaviors and impact patient care, and self-assessment may be unreliable. While students reported considering cost and impact of tests prior to ordering them, they are not the ordering providers at this stage of their training. Changing ordering practices may be more feasible if students on their clinical rotations feel empowered to discuss HVC concepts with residents and attendings. Given prior studies that show a deficiency in faculty and resident understanding of HVC,[21,38,39] ongoing efforts for faculty development in this area should be continued. However, we hoped that this workshop would give students the tools to initiate discussions with both senior members of the team and patients/families. We also hoped that the information learned in this session carries beyond medical school and will be incorporated into these students' future practice. Patients, the hospital system, and the US health care system as a whole may benefit from increased awareness of the financial aspects of health care delivery.

Appendices

A. Facilitator Guide.docx
B. Clinical Vignette.docx
C. PowerPoint Presentation.pptx
D. Cost List.xlsx
E. Role-Play Cases.docx
F. Pre- and Postsurvey.docx

All appendices are peer reviewed as integral parts of the Original Publication.

Priya N. Jain, MD: Assistant Professor, Department of Pediatrics, Children's Hospital at Montefiore, Albert Einstein College of Medicine

Steven Eagle, MD: Nemours Alfred I duPont Hospital for Children

Miriam Schechter, MD: Associate Professor, Department of Pediatrics, Children's Hospital at Montefiore, Albert Einstein College of Medicine

Copyright © 2020 Jain et al. This is an open-access publication distributed under the terms of the Creative Commons Attribution license.

Hai Jung H. Rhim, MD: Assistant Professor, Department of Pediatrics, Children's Hospital at Montefiore, Albert Einstein College of Medicine

Rhonda Acholonu, MD: Assistant Professor, Department of Pediatrics, Children's Hospital at Montefiore, Albert Einstein College of Medicine

Acknowledgments
We would like to thank Gabriella Azzarone, MD, Aileen Mendez, and Patricia Hametz, MD.

Disclosures
None to report.

Funding/Support
None to report.

Ethical Approval
The Albert Einstein College of Medicine Institutional Review Board approved this study.

References

1. Institute of Medicine Roundtable on Evidence-Based Medicine, Yong PL, Saunders RS, Olsen L, eds. *The Healthcare Imperative: Lowering Costs and Improving Outcomes*. National Academies Press; 2010. https://doi.org/10.17226/12750
2. Keehan SP, Stone DA, Poisal JA, et al. National health expenditures projections, 2016-25: price increases, aging push sector to 20 percent of economy. *Health Aff (Millwood).* 2017;36(3):553-563. https://doi.org/10.1377/hlthaff.2016.1627
3. Berwick DM, Hackbarth AD. Eliminating waste in US health care. *JAMA*. 2012;307(14):1513-1516. https://doi.org/10.1001/jama.2012.362
4. Cook DA, Beckman TJ. High-value, cost-conscious medical education. *JAMA Pediatr*. 2015;169(2):109-111. https://doi.org/10.1001/jamapediatrics.2014.2964
5. Nelson DE, Thompson BL, Bland SD, Rubinson R. Trends in perceived cost as a barrier to medical care, 1991-1996. *Am J Public Health.* 1999;89(9):1410-1413. https://doi.org/10.2105/AJPH.89.9.1410
6. Nipp RD, Powell E, Chabner B, Moy B. Recognizing the financial burden of cancer patients in clinical trials. *Oncologist*. 2015; 20(6):572-575. https://doi.org/10.1634/theoncologist.2015-0068
7. Cooke M. Cost consciousness in patient care—what is medical education's responsibility? *N Engl J Med*. 2010;362(14): 1253-1255. https://doi.org/10.1056/NEJMp0911502
8. Johnson DP, Browning WL, Gay JC, Williams DJ. Pediatric hospitalist perceptions regarding trainees' effects on cost and quality of care. *Hosp Pediatr*. 2015;5(4):211-218. https://doi.org/10.1542/hpeds.2014-0086
9. Weinberger SE. Providing high-value, cost-conscious care: a critical seventh general competency for physicians. *Ann Intern Med*. 2011;155(6):386-388. https://doi.org/10.7326/0003-4819-155-6-201109200-00007
10. Oppong R, Mistry H, Frew E. Health economics education in undergraduate medical training: introducing the health economics education (HEe) website. *BMC Med Educ*. 2013;13:126. https://doi.org/10.1186/1472-6920-13-126
11. Steele C, Cayea D, Berk J, et al. Novel first-year curriculum in high-value care. *Clin Teach*. 2019;16(5):513-518. https://doi.org/10.1111/tct.12989
12. Choosing Wisely: Promoting Conversations Between Patients and Clinicians. American Board of Internal Medicine Foundation. Accessed September 30, 2020. http://www.choosingwisely.org
13. Cassel CK, Guest JA. Choosing wisely: helping physicians and patients make smart decisions about their care. *JAMA*. 2012; 307(17):1801-1802. https://doi.org/10.1001/jama.2012.476
14. Smith CD, Alliance for Academic Internal Medicine—American College of Physicians High Value, Cost-Conscious Care Curriculum Development Committee. Teaching high-value, cost-conscious care to residents: the Alliance for Academic Internal Medicine—American College of Physicians Curriculum. *Ann Intern Med*. 2012;157(4):284-286. https://doi.org/10.7326/0003-4819-157-4-201208210-00496
15. Tchou MJ, Walz A, Burgener E, Schroeder A, Blankenburg R. Teaching high-value care in pediatrics: a national survey of current practices and guide for future curriculum development. *J Grad Med Educ*. 2017;9(6):741-747. https://doi.org/10.4300/JGME-D-17-00139.1
16. Hamburger S. Cost effectiveness in an internal medicine residency program: one physician's approach. *South Med J*. 1982;75(4):429-433. https://doi.org/10.1097/00007611-198204000-00013
17. Sommers BD, Desai N, Fiskio J, et al. An educational intervention to improve cost-effective care among medicine housestaff: a randomized controlled trial. *Acad Med*. 2012;87(6):719-728. https://doi.org/10.1097/ACM.0b013e31825373b3
18. Avery CS, Beck J, Padrez R, et al. Developing the PedsValue Modules—a national pediatric high value care curriculum. *Acad Pediatr*. 2017;17(5):471-475. https://doi.org/10.1016/j.acap.2017.04.006
19. Weinberger SE. Educating trainees about appropriate and cost-conscious diagnostic testing. *Acad Med*. 2011;86(11):1352. https://doi.org/10.1097/ACM.0b013e3182308db7
20. Hernu R, Cour M, de la Salle S, Robert D, Argaud L, Costs in French ICU Study Group. Cost awareness of physicians in intensive care units: a multicentric national study. *Intensive Care Med*. 2015;41(8):1402-1410. https://doi.org/10.1007/s00134-015-3859-1
21. Rock TA, Xiao R, Fieldston E. General pediatric attending physicians' and residents' knowledge of inpatient hospital finances. *Pediatrics*. 2013;131(6):1072-1080. https://doi.org/10.1542/peds.2012-1753

Copyright © 2020 Jain et al. This is an open-access publication distributed under the terms of the Creative Commons Attribution license.

22. Cayea D, Tartaglia K, Pahwa A, Harrell H, Shaheen A, Lang VJ. Current and optimal training in high-value care in the internal medicine clerkship: a national curricular needs assessment. *Acad Med*. 2018;93(10):1511-1516. https://doi.org/10.1097/ACM.0000000000002192

23. Woods S, Avery C, Bartlett K, et al. High-value care pediatric curriculum. *MedEdPORTAL*. 2015;11:10146. https://doi.org/10.15766/mep_2374-8265.10146

24. Walker L, Whitney S, Rama J. Teaching high value care to pediatric residents using an online, interactive, case-based module: does it translate to change? *Pediatrics*. 2018;141 (1 MeetingAbstract):437. https://doi.org/10.1542/peds.141.1_MeetingAbstract.437

25. Walker LL, Hopkins A, Rooholamini SN, et al. Teaching high-value care: case vignettes for pediatric practice. *MedEdPORTAL*. 2018; 14:10723. https://doi.org/10.15766/mep_2374-8265.10723

26. Blackwell M, Moore K, Kocoloski A, Gill P. Teaching and evaluating high-value care through a novel case-based morning report curriculum. *MedEdPORTAL*. 2016;12:10356. https://doi.org/10.15766/mep_2374-8265.10356

27. Dewan M, Herrmann LE, Tchou MJ, et al. Development and evaluation of high-value pediatrics: a high-value care pediatric resident curriculum. *Hosp Pediatr*. 2018;8(12):785-792. https://doi.org/10.1542/hpeds.2018-0115

28. Iscoe M, Lord R, Schulz J, Lee D, Cayea D, Pahwa A. Teaching medical students about cost-effectiveness. *Clin Teach*. 2018;15(1):24-28. https://doi.org/10.1111/tct.12613

29. Aagaard E, Wagner R, Jackson D, Earnest M. Teaching the cost of hospital care to medical students. *MedEdPORTAL*. 2010;6:7787. https://doi.org/10.15766/mep_2374-8265.7787

30. Cooperstein SE, Kocevar-Weidinger E. Beyond active learning: a constructivist approach to learning. *Ref Serv Rev*. 2004;32(2): 141-148. https://doi.org/10.1108/00907320410537658

31. Bonwell CC, Eison JA. *Active Learning: Creating Excitement in the Classroom*. The George Washington University; 1991.

32. Graffam B. Active learning in medical education: strategies for beginning implementation. *Med Teach*. 2007;29(1):38-42. https://doi.org/10.1080/01421590601176398

33. Le H, Janssen J, Wubbels T. Collaborative learning practices: teacher and student perceived obstacles to effective student collaboration. *Cambridge J Educ*. 2018;48(1):103-122. https://doi.org/10.1080/0305764X.2016.1259389

34. Healthcare Bluebook. Accessed August 1, 2019. https://www.healthcarebluebook.com/

35. Bhanji F, Gottesman R, de Grave W, Steinert Y, Winer LR. The retrospective pre-post: a practical method to evaluate learning from an educational program. *Acad Emerg Med*. 2012;19(2): 189-194. https://doi.org/10.1111/j.1553-2712.2011.01270.x

36. Drennan J, Hyde A. Controlling response shift bias: the use of the retrospective pre-test design in the evaluation of a master's programme. *Assess Eval High Educ*. 2008;33(6):699-709. https://doi.org/10.1080/02602930701773026

37. Levinson W, Gordon G, Skeff K. Retrospective versus actual pre-course self-assessments. *Eval Health Prof*. 1990;13(4): 445-452. https://doi.org/10.1177/016327879001300406

38. Jonas JA, Ronan JC, Petrie I, Fieldston ES. Description and evaluation of an educational intervention on health care costs and value. *Hosp Pediatr*. 2016;6(2):72-79. https://doi.org/10.1542/hpeds.2015-0138

39. Patel MS, Reed DA, Smith C, Arora VM. Role-modeling cost-conscious care—a national evaluation of perceptions of faculty at teaching hospitals in the United States. *J Gen Intern Med*. 2015;30(9):1294-1298. https://doi.org/10.1007/s11606-015-3242-5

Received: February 7, 2020
Accepted: July 2, 2020
Published: November 17, 2020

Copyright © 2020 Jain et al. This is an open-access publication distributed under the terms of the Creative Commons Attribution license.
